# Virome Analysis Provides an Insight into the Viral Community of Chinese Mitten Crab *Eriocheir sinensis*

**DOI:** 10.1128/spectrum.01439-23

**Published:** 2023-06-26

**Authors:** Guangyu Guo, Muhua Wang, Dandan Zhou, Xinyi He, Peiyun Han, Gongrui Chen, Jiamin Zeng, Zhi Liu, Yinqing Wu, Shaoping Weng, Jianguo He

**Affiliations:** a State Key Laboratory of Biocontrol, School of Marine Sciences, Sun Yat-sen University, Zhuhai, China; b China-ASEAN Belt and Road Joint Laboratory on Mariculture Technology, Southern Marine Science and Engineering Guangdong Laboratory (Zhuhai), Zhuhai, China; c Guangdong Province Key Laboratory for Aquatic Economic Animals, School of Life Sciences, Sun Yat-sen University, Guangzhou, China; Changchun Veterinary Research Institute

**Keywords:** *Eriocheir sinensis*, virome, virus taxonomy, milky disease, hepatopancreatic necrosis syndrome

## Abstract

Recent advances in viromics have led to the discovery of a great diversity of RNA viruses and the identification of a large number of viral pathogens. A systematic exploration of viruses in Chinese mitten crab (Eriocheir sinensis), one of the most important aquatic commercial species, is still lacking. Here, we characterized the RNA viromes of asymptomatic, milky disease (MD)-affected, and hepatopancreatic necrosis syndrome (HPNS)-affected Chinese mitten crabs collected from 3 regions in China. In total, we identified 31 RNA viruses belonging to 11 orders, 22 of which were first reported here. By comparing viral composition between samples, we observed high variation in viral communities across regions, with most of the viral species being region-specific. We proposed to establish several novel viral families or genera based on the phylogenetic relationships and genome structures of viruses discovered in this study, expanding our knowledge of viral diversity in brachyuran crustaceans.

**IMPORTANCE** High-throughput sequencing and meta-transcriptomic analysis provide us with an efficient tool to discover unknown viruses and explore the composition of viral communities in specific species. In this study, we investigated viromes in asymptomatic and diseased Chinese mitten crabs collected from three distant locations. We observed high regional variation in the composition of viral species, highlighting the importance of multi-location sampling. In addition, we classified several novel and ICTV-unclassified viruses based on their genome structures and phylogenetic relationships, providing a new perspective on current viral taxa.

## INTRODUCTION

Viruses are the most abundant and diverse biological entities on earth. The virome, the assemblage of viruses associated with a particular ecological niche or organism, is vast and heterogeneous (1). However, the nature and structure of viral communities were mostly unappreciated until the development of high-throughput sequencing techniques and metagenomic analysis approaches. Many novel viruses, especially RNA viruses, have been described and characterized through virome analysis using next-generation sequencing (2–6). Virome analysis can detect DNA and RNA viruses with high sensitivity and low stochastic error derived from the relative abundance of viromic taxa (7, 8). Therefore, virome analysis has been widely applied in human, animal, and environmental samples to detect novel viruses and investigate the composition of viral populations (1, 9–15).

Viruses are found in all types of organisms and their community states can be associated with adverse outcomes for their hosts (1). Viral infection causes multiple physiological and behavioral symptoms and even death in brachyuran crustaceans (16, 17). For example, infection with Scylla serrata reovirus SZ-2007 (also called “mud crab virus,” MCRV) leads to a high mortality rate of up to 100% in mud crabs (Scylla serrata) (18). The analysis of viromes can also be used to uncover potential pathogens and monitor the distribution of viral pathogens (12, 19–21). However, although multi-location sampling is common in viromic studies for both the discovery of novel viruses and the surveillance of viral pathogens (4, 12, 15, 20, 22), few studies have investigated how viral species composition in the same animal species varies in different locations.

The Chinese mitten crab, Eriocheir sinensis H. Milne Edwards 1853, is one of the most important aquatic commercial species. The freshwater aquaculture production of Chinese mitten crab makes up 6.9% of global crustacean aquaculture production (23). The incidence of disease outbreaks has increased with the expanding aquaculture industry of Chinese mitten crab. “Milky disease” (MD), named after the milky hemolymph of sick crabs, causes clinical signs in infected crabs, including weakness, opaque or whitish muscles, milky hemolymph, and subsequent death due to organ failure (24). Another disease affecting crabs, called hepatopancreatic necrosis syndrome (HPNS, also called hepatopancreatic necrosis disease), causes multiple symptoms, including a weak response to stimulation, reduced preening activity and locomotion, muscle atrophy and edema, empty stomachs and intestines, and white focal patches in the hepatopancreas (25, 26). The mortality rates of MD and HPNS are >20% and >40%, respectively (24, 25). The epidemic of MD and HPNS has significantly injured the Chinese mitten crab aquaculture industry and led to marked economic loss.

Here, we investigated the RNA viromes in pooled samples of MD-affected, HPNS-affected, and asymptomatic Chinese mitten crabs collected from three regions in China. We compared and investigated the viral composition in crabs from different regions, as well as the relative abundance of viral species between samples from diseased and asymptomatic crabs. Additionally, we proposed to establish several novel families and genera and reconstruct the taxonomy of specific families based on phylogenetic analyses of newly discovered viruses. Our results reveal a great diversity of viruses in Chinese mitten crabs and expand our knowledge of viral communities in crabs.

## RESULTS

### Virome overview.

**FIG 1:**
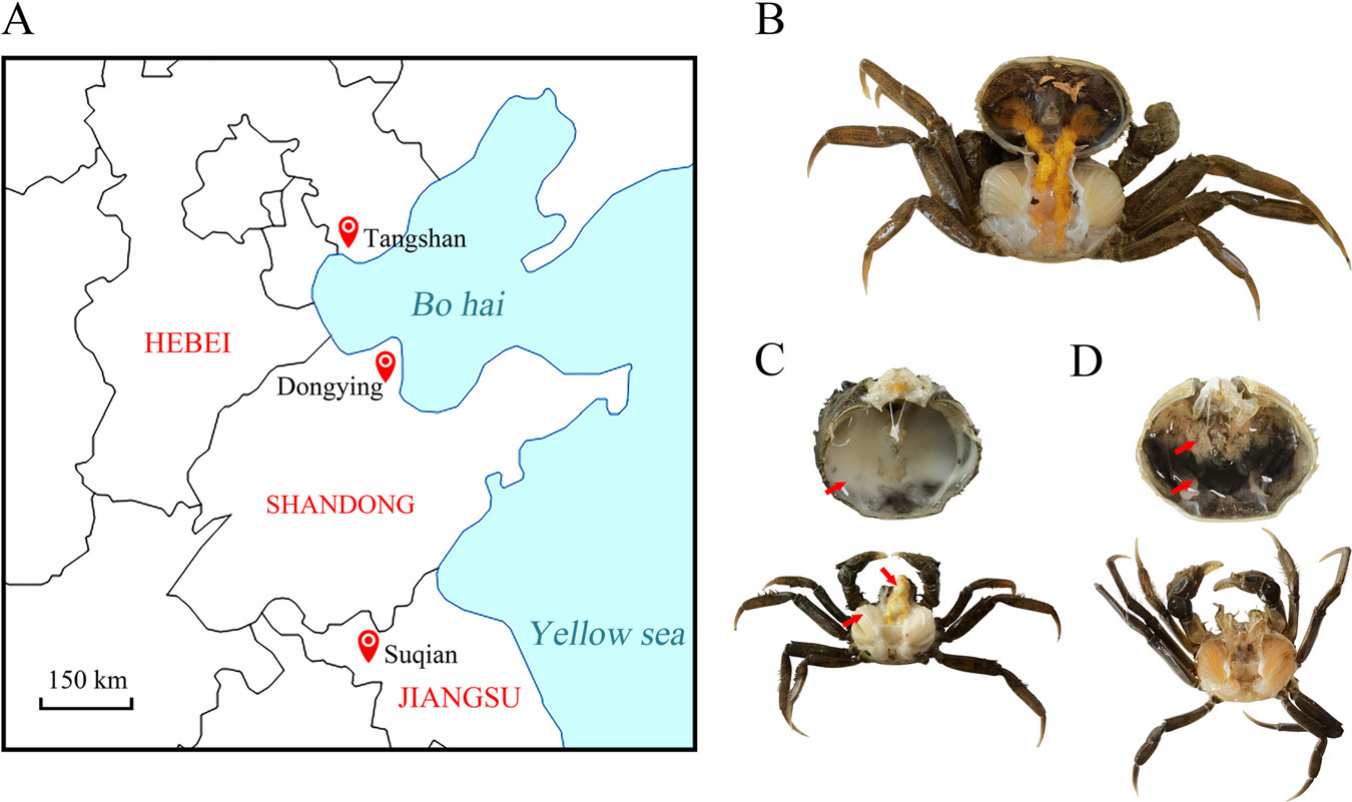
Sampling locations and conditions of crab samples. (A) Map of sampling locations. (B) Asymptomatic crab. (C) Symptoms of MD-infected crab. Arrows indicate the milky hemolymph and albescent hepatopancreas of diseased crab. (D) Symptoms of HPNS-infected crab. Arrows indicate atrophic hepatopancreas and transparent hemolymph.

Sixty diseased or asymptomatic Chinese mitten crabs collected from three regions of China were divided into 6 pools based on their health status and sampling locations (Fig. 1A; Table 1). We identified crabs with MD based on clinical symptoms, including milky hemolymph filling the carapace and gills, albescent hepatopancreas, and inactive behavior (Fig. 1C). Crabs with atrophic hepatopancreas and muscles, transparent hemolymph, and inactive behavior were identified as having HPNS (Fig. 1D). To increase the viral abundance, the hepatopancreas, gills, and muscles of 10 crabs with MD (TS-MD) and 10 asymptomatic crabs (TS-Asym) collected from Tangshan, Hebei Province were pooled to construct sequencing libraries. In addition, sequencing libraries were constructed using the hepatopancreas, gills, and muscles of 10 crabs with HPNS (DY-HPNS) and 10 asymptomatic crabs (DY-Asym) from Dongying, Shandong Province. The hepatopancreas, gills, and muscles of 10 crabs with HPNS (SQ-HPNS) and 10 asymptomatic crabs (SQ-Asym) collected from Suqian, Jiangsu Province, were also pooled to construct sequencing libraries.

**TABLE 1:** Overview of 6 sequencing pools^*a*^

Condition	Sampling		Crabs (*n*)	RNA library	Raw		Clean	
	Location	Date (mo/day/year)			Bases (Gb)	Reads (M)	Bases (Gb)	Reads (M)
Milky disease	Tangshan	4/18/2021	10	TS-MD	10.71	71.40	9.65	69.50
Asymptomatic	Tangshan	4/18/2021	10	TS-Asym	11.91	79.43	11.05	77.20
HPNS	Dongying	8/13/2021	10	DY-HPNS	11.78	78.53	10.10	76.11
Asymptomatic	Dongying	8/13/2021	10	DY-Asym	10.53	70.19	9.30	68.41
HPNS	Suqian	10/22/2021	10	SQ-HPNS	25.10	167.35	19.39	159.98
Asymptomatic	Suqian	10/22/2021	10	SQ-Asym	10.74	71.62	8.20	68.76

a MD, milky disease; HPNS, hepatopancreatic necrosis syndrome; TS, Tangshan (Hebei Province); DY, Dongying (Shandong Province); SQ, Suqian (Jiangsu Province).

**FIG 2:**
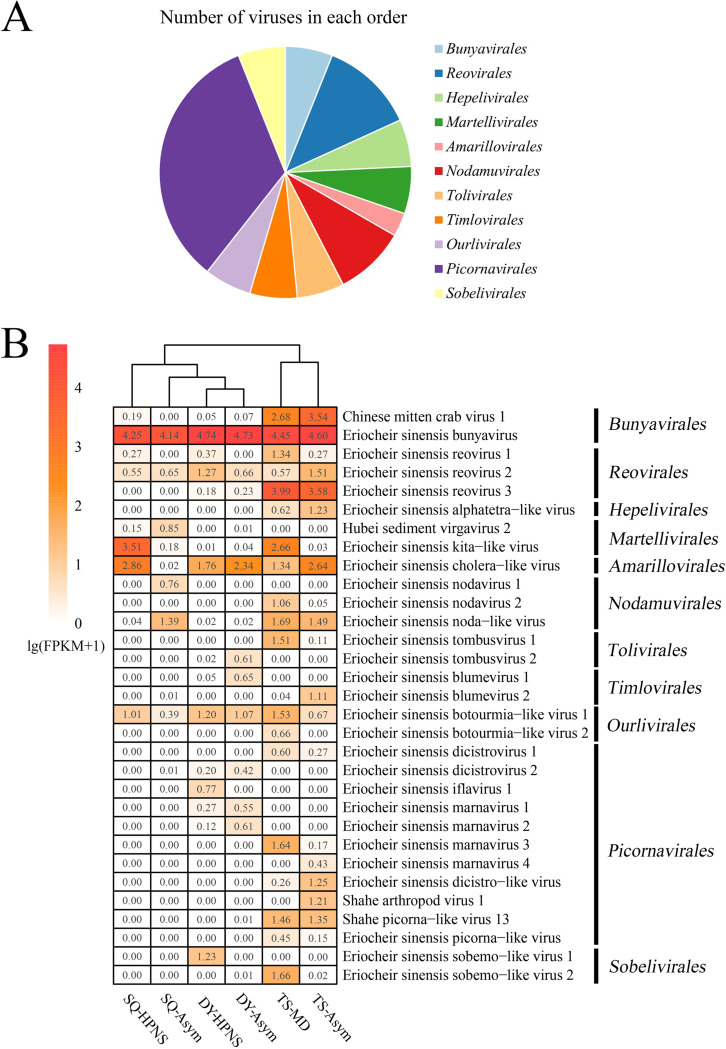
Overview of the RNA virome in Chinese mitten crab. (A) Number of RNA viruses in viral orders. (B) Abundance of viruses in each library. The relative abundance of viruses in each library was calculated and normalized by the number of fragments per kilobase of exon per million fragments mapped (FPKM).

In total, 80.77 Gb of Illumina reads were generated for 6 RNA sequencing libraries. A total of 483,997 contigs were obtained by *de novo* assembling the trimmed Illumina reads from each library, with 394 of them identified as viral sequences. We found 31 viruses from these 394 viral contigs (Table 2). These RNA viruses belonged to the orders *Reovirales*, *Bunyavirales*, *Hepelivirales*, *Martellivirales*, *Amarillovirales*, *Nodamuvirales*, *Tolivirales*, *Timlovirales*, *Ourlivirales*, *Picornavirales*, and *Sobelivirales* based on their sequence similarity, genome structure, and phylogenetic relationships (Fig. 2). Notably, 22 of these 31 viruses are reported for the first time here. The genomes of 4 viruses were assembled more completely in this study than in previous reports.

**TABLE 2:** Viruses identified in this study

Virus name	GenBank accession no.	Genome type	Genome length (bp)	Order	Family
Eriocheir sinensis reovirus 1	OP019112–OP019124	dsRNA	20,180	*Reovirales*	*Sedoreoviridae*
Eriocheir sinensis reovirus 2	OP019125	dsRNA	4,486	*Reovirales*	*Spinareoviridae*
Eriocheir sinensis reovirus 3	KP638402–KP638413	dsRNA	23,913	*Reovirales*	*Sedoreoviridae*
Chinese mitten crab virus 1	MH717874–MH717876	–ssRNA	10,793	*Bunyavirales*	*Cruliviridae*
Eriocheir sinensis bunyavirus	OP019095	–ssRNA	12,238	*Bunyavirales*	
Eriocheir sinensis alphatetra-like virus	OP019089	+ssRNA	10,673	*Hepelivirales*	
Hubei sediment virgavirus 2	MW897259	+ssRNA	6,916	*Martellivirales*	*Virgaviridae*
Eriocheir sinensis kita-like virus	OP019101	+ssRNA	12,933	*Martellivirales*	
Eriocheir sinensis cholera-like virus	OP019096	+ssRNA	18,830	*Amarillovirales*	*Flaviviridae*
Eriocheir sinensis nodavirus 1	OP019107	+ssRNA	3,087	*Nodamuvirales*	*Nodaviridae*
Eriocheir sinensis nodavirus 2	OP019108	+ssRNA	3,018	*Nodamuvirales*	*Nodaviridae*
Eriocheir sinensis noda-like virus	OP019106	+ssRNA	4,802	*Nodamuvirales*	
Eriocheir sinensis tombusvirus 1	OP019131	+ssRNA	4,861	*Tolivirales*	*Tombusviridae*
Eriocheir sinensis tombusvirus 2	OP019132	+ssRNA	4,283	*Tolivirales*	*Tombusviridae*
Eriocheir sinensis blumevirus 1	OP019090	+ssRNA	5,266	*Timlovirales*	*Blumeviridae*
Eriocheir sinensis blumevirus 2	OP019091	+ssRNA	3,514	*Timlovirales*	*Blumeviridae*
Eriocheir sinensis botourmia-like virus 1	OP019092	+ssRNA	3,493	*Ourlivirales*	*Botourmiaviridae*
Eriocheir sinensis botourmia-like virus 2	OP019093	+ssRNA	3,404	*Ourlivirales*	*Botourmiaviridae*
Eriocheir sinensis dicistrovirus 1	OP019098	+ssRNA	7,577	*Picornavirales*	*Dicistroviridae*
Eriocheir sinensis dicistrovirus 2	OP019099	+ssRNA	7,116	*Picornavirales*	*Dicistroviridae*
Eriocheir sinensis dicistro-like virus	OP019097	+ssRNA	9,308	*Picornavirales*	
Shahe arthropod virus 1	KX883988	+ssRNA	9,289	*Picornavirales*	
Eriocheir sinensis iflavirus 1	OP019100	+ssRNA	3,596	*Picornavirales*	*Iflaviridae*
Eriocheir sinensis marnavirus 1	OP019102	+ssRNA	8,832	*Picornavirales*	*Marnaviridae*
Eriocheir sinensis marnavirus 2	OP019103	+ssRNA	8,634	*Picornavirales*	*Marnaviridae*
Eriocheir sinensis marnavirus 3	OP019104	+ssRNA	8,142	*Picornavirales*	*Marnaviridae*
Eriocheir sinensis marnavirus 4	OP019105	+ssRNA	7,809	*Picornavirales*	*Marnaviridae*
Shahe picorna-like virus 13	KX883657	+ssRNA	9,077	*Picornavirales*	
Eriocheir sinensis picorna-like virus	OP019111	+ssRNA	8,297	*Picornavirales*	
Eriocheir sinensis sobemo-like virus 1	OP019126	+ssRNA	3,124	*Sobelivirales*	
Eriocheir sinensis sobemo-like virus 2	OP019127	+ssRNA	3,046	*Sobelivirales*	

To verify the presence of RNA-dependent RNA polymerase (RdRp) genes, we used the original samples for reverse transcription-PCR (RT-PCR) and nested RT-PCR with primer sets designed based on the RdRp sequences of viral genomes (Table S1 in the supplemental material). The products of RT-PCR were confirmed by Sanger sequencing. To ensure that our assembled viral genomes were not chimeric sequences, we aligned filtered Illumina reads to the genomes and calculated the genome coverage of reads as well as the heterozygosity of single-nucleotide polymorphisms (SNPs). The extremely high genome coverage (>99.5%) and low heterozygosity (<0.5%) indicate that our assemblies were not chimeric sequences and were high-quality (Table S2).

### Variations of viral composition among samples.

**FIG 3:**
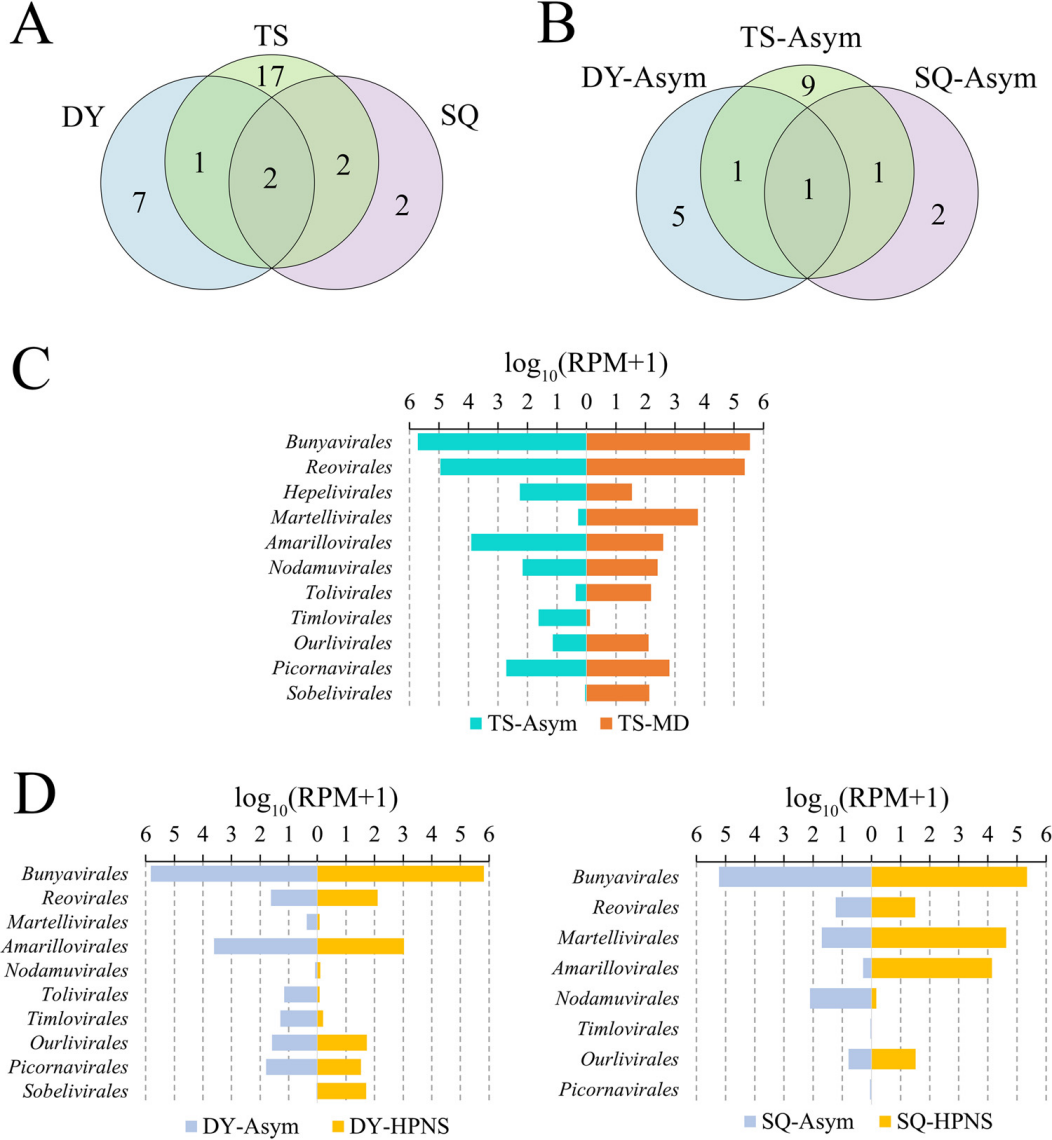
Variation in virus composition among sampling locations and crab conditions. (A) Venn plot of the number of RNA viruses in different sampling locations. (B) Venn plot of the number of RNA viruses in asymptomatic crabs collected from three sampling locations. (C) Comparison of relative abundance of viral orders in asymptomatic and MD-infected crabs collected from Tangshan, Hebei Province. (D) Comparison of relative abundance of viral orders in asymptomatic and HPNS-infected crabs collected from Tangshan, Hebei Province and Suqian, Jiangsu Province. Relative abundance of viral orders in each library was calculated and normalized by the number of mapped reads per million total reads (RPM).

There was large variation in the virus composition in Chinese mitten crab collected from the three sampling locations, which are separated from each other by at least 200 km (Fig. 3A and B). Most of the viruses (26/31, 80.6%) were region-specific. Eriocheir sinensis bunyavirus (EsBV) was found in the sequencing libraries of all sampling locations at high abundance, making up more than 70% of the viral reads in each library (Fig. 2B).

The relative abundance of viruses was compared between diseased and asymptomatic crab samples. The relative abundance of 13 viruses was higher in MD-affected crabs than in asymptomatic crabs collected in Tangshan (Fig. 2B). The orders *Martellivirales*, *Tolivirales*, *and Sobelivirales* were abundant in MD-affected crabs, but showed a very low relative abundance in asymptomatic crabs (Fig. 3C). Six viruses showed a higher relative abundance in HPNS-affected crabs than in asymptomatic crabs collected in Dongying, and 6 viruses showed a higher relative abundance in HPNS-affected crabs than in asymptomatic crabs in Suqian (Fig. 2B). In the crabs with HPNS, only the order *Sobelivirales* was much more abundant than in asymptomatic crabs in the samples collected from Dongying, and the orders *Amarillovirales* and *Martellivirales* were much more abundant in HPNS-affected crabs than in asymptomatic crabs in the samples collected from Suqian (Fig. 3D). No viruses were found to have higher abundance in HPNS-affected crabs than in asymptomatic crabs from both Dongying and Suqian, suggesting that viruses may not be the main cause of HPNS.

### Double-stranded RNA virus.

Three double-stranded RNA (dsRNA) viruses found in this study belonged to the order *Reovirales*. Eriocheir sinensis reovirus isolate 905 (EsRV905) and Eriocheir sinensis reovirus isolate 816 (EsRV816) were both first reported in 2002 (27–29). Eriocheir sinensis reovirus strain WX-2012 (EsRV WX-2012) was first reported in 2015 (30). These three viruses were all named Eriocheir sinensis reovirus (EsRV) but showed no significant similarity with each other. Only EsRV905 has been accepted as a viral species of the genus *Cardoreovirus*, family *Sedoreoviridae*, by the International Committee on Taxonomy of Viruses (ICTV) (31). EsRV816 and EsRV WX-2012 have been proposed to be classified into the two novel genera “Crustareovirus” and “Carbreovirus,” respectively (32). Our phylogenetic result supported this proposal (Fig. S1). To prevent researchers from mistaking them for the same species, we propose to retain ESRV isolate 915 as Eriocheir sinensis reovirus, but rename ESRV isolate 816 to Eriocheir sinensis reovirus 2 (EsRV2) and ESRV strain WX-2012 to Eriocheir sinensis reovirus 3 (EsRV3).

Incomplete genome segments encoding part of RdRp have been previously reported for EsRV (GenBank accession no. AY542965) and EsRV2 (EF508130). We assembled a more complete genome of EsRV that consisted of 12 segments. The fifth segment of EsRV was not fully assembled, causing this segment to be divided into two fragments. EsRV was closely related to Callinectes sapidus reovirus 2 (CsRV2, GenBank accession no. MW208677 to MW208688). A previous study proposed that the genome of EsRV2 had 10 segments, but only a complete segment encoding RdRp (4,488 bp) was found in our assembled contigs (29).

### Negative-sense single-stranded RNA virus.

**FIG 4:**
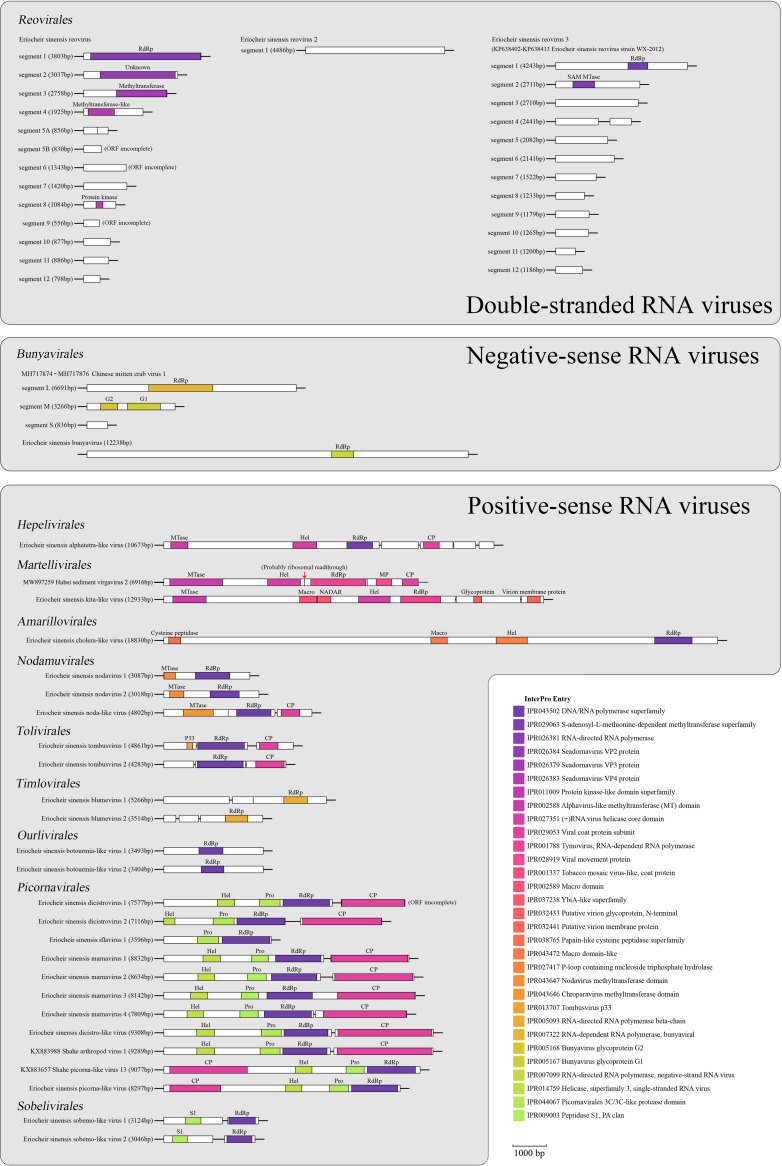
Genome organization of viruses identified in this study. Genome organization of 31 viruses found in this study is shown. Predicted regions that encode major functional proteins or domains are labeled with color boxes. CP, coat protein; Hel, helicase; MP, movement protein; MTase, methyltransferase; NADAR, NAD and ADP-ribose; Pro, protease; RdRp, RNA-dependent RNA polymerase.

Two negative-sense single-stranded RNA (ssRNA) viruses belonging to the order *Bunyavirales* were identified in this study. Chinese mitten crab virus 1 (GenBank accession no. MH717874 to MH717876), which was reported in 2019 and accepted by ICTV as a member of the family *Cruliviridae* in order *Bunyavirales* in 2020, was found in pools TS-MD and TS-Asym (33, 34). A novel bunyavirus sequence (12,238 bp in length), Eriocheir sinensis bunyavirus (EsBV), was discovered and dominated in all 6 sampling pools. Most of the viral families within *Bunyavirales* have genomes composed of multiple linear single-stranded segments (2 to 8 segments), except for members of the new family *Tulasviridae* which have a genome composed of a single large molecule of negative-sense RNA (~12 kb) encoding three open reading frames (ORFs) (35, 36). However, EsBV has a non-segmented genome of more than 12,000 nucleotides (nt) in length with only one ORF containing a predicted RdRp domain (Fig. 4), unlike the genome organization of other families in *Bunyavirales*. A phylogenetic tree constructed using the conserved RdRp domain showed that EsBV, SBlV3, and SBlV4 clustered together and formed a divergent clade with a long branch, sister to the families *Wupedeviridae* and *Nairoviridae* (Fig. S2). EsBV shared 25.7% amino acid identity with Shahe bunya-like virus 3 (SBlV3) and 24.9% with Shahe bunya-like virus 4 (SBlV4). The unique genome organization of EsBV and its low sequence similarity with SBlV3 and SBlV4 suggested that EsBV is a novel virus. The phylogenetic result and distinct genome organization of EsBV indicated the possibility of establishing a novel family in the order *Bunyavirales*. However, there are some difficulties in the establishment of this novel family, mainly because EsBV and its relatives have no obvious common features, and further taxonomic research is needed in the future.

### Positive-sense ssRNA virus.

Twenty-six positive-sense ssRNA viruses belonging to the orders *Hepelivirales*, *Martellivirales*, *Amarillovirales*, *Nodamuvirales*, *Tolivirales*, *Timlovirales*, *Ourlivirales*, *Picornavirales*, and *Sobelivirales* were identified in this study. Many of the viruses we found have different genome organizations compared to current ICTV-accepted members and independently clustered outside present genera or families in our phylogenetic trees. For these viruses, we preliminarily propose to establish novel genera or families mainly based on their phylogenetic results and genome organization.

**(i) Virus in the order *Hepelivirales*.** A viral sequence related to the family *Alphatetraviridae* in the order *Hepelivirales*, tentatively named Eriocheir sinensis alphatetra-like virus (EsAlV), was mainly discovered in the pool TS-Asym. EsAlV possessed a non-segmented genome with 10,673 nt encoding five ORFs (Fig. 4), different from the genome organization of four families of *Hepelivirales* (37–40). The 5′ ORF was longest and predicted to contain a methyltransferase domain, helicase domain, and RdRp domain. Four 3′-proximal ORFs (ORFs 2 to 5) were relatively short, and ORF3 was predicted to encode a coat protein domain. Phylogenetic analysis using the RdRp revealed that EsAlV formed a clade with previously described unclassified viruses that was sister to the family *Alphatetraviridae* (Fig. S3). EsAlV shared 44.3% amino acid identity with Shahe hepe-like virus 2, which was the most closely related virus to EsAlV, indicating that EsAlV was a novel virus. The distinct genome structure of EsAlV and the long branch length of the clade containing EsAlV suggested that EsAlV may belong to a novel family in the order *Hepelivirales*. We propose to establish a novel family named “Pcahviridae” in *Hepelivirales*. This name was derived from the contraction of its characteristics, being a polycistronic, aquatic, hepe-like virus.

**(ii) Viruses in the order *Martellivirales*.** A kita-like viral sequence 12,933 bp in length, tentatively named Eriocheir sinensis kita-like virus (EsKlV), was discovered in this study. Current members of the family *Kitaviridae* have multiple segments (2 to 4 segments) and were considered to affect plants (41). Phylogenetic analysis revealed that EsKlV clustered with Sanxia atyid shrimp virus 1 (SASV1) and Beihai anemone virus 1 (BAV1) and fell within the family *Kitaviridae* (Fig. S4). EsKlV shared low amino acid identities with SASV1 (26.3%) and BAV1 (36.2%), suggesting that it may be a novel viral species in *Kitaviridae*. However, EsKlV was monopartite, and its genome contained 3 ORFs encoding 7 types of domains in total (Fig. 4). EsKlV and its closely related viruses BAV1 and Erysiphe necator associated virga-like virus 1 showed multiple host types, including crustaceans, anthozoans, and fungi. The genome organization and host range of EsKlV were significantly distinct from those of current members of *Kitaviridae*. Previous studies have found a number of invertebrate-infecting viruses closely related to members of *Kitaviridae* and suggested that members of *Kitaviridae* may primarily be arthropod/invertebrate viruses capable of limited spread in plant tissues, because most kitaviruses produce non-systemic infection (41–45). The discovery of EsKlV and its relatives reinforced the concept that *Kitaviridae* may be a family of invertebrate-infecting viruses. Additionally, based on the distinct genome organization and phylogenetic result, we propose to place EsKlV and its similar viruses in the new genus “Monokitavirus” in *Kitaviridae*. This name was derived because EsKlV is a monopartite kita-like virus.

**(iii) Viruses in the order *Amarillovirales*.** *Flaviviridae* is a family of positive-sense RNA viruses that have non-segmented genomes of approximately 9 to 13 kb and affect mammals (46). A partial genome of Eriocheir sinensis cholera-like virus (EsClV, GenBank accession no. KM405246, 2,018 bp) closely related to members of the family *Flaviviridae*, order *Amarillovirales*, was reported in 2016. Here, we found EsClV in almost all sampling pools except for pool SQ-Asym (Fig. 2B) and generated a more complete genome sequence of EsClV with a length of 18,830 bp (Fig. 3). EsClV has a genome organization with a single ORF containing a papain-like cysteine peptidase domain, macro domain, helicase domain, and RdRp domain (Fig. 4). Phylogenetic analysis revealed that EsClV was closely related to a few viral sequences with genome lengths up to ~24,500 nt (up to 40.4% amino acid identity), and they formed a distinct clade that was not clustered within current genera of *Flaviviridae* (Fig. S5). The differences in genome length and host range between EsClV and current members of *Flaviviridae* indicate that EsClV and its relatives may represent a novel family in the order *Amarillovirales*. We propose to name this family “*Megamonornaviridae*.” This name was derived because these viruses have a large genome, are monopartite, and have a single-stranded RNA genome. Further study of the common characteristics of “*Megamonornaviridae*” needs to be performed to establish the novel family in the future.

**(iv) Viruses in the order *Nodamuvirales*.** In our work, three novel viral sequences belonging to the order *Nodamuvirales* were identified, tentatively named Eriocheir sinensis nodavirus 1 (EsNV1), Eriocheir sinensis nodavirus 2 (EsNV2), and Eriocheir sinensis noda-like virus (EsNlV). Members of the family *Nodaviridae* have two genomic RNAs, encoding the RdRp (~3.1 kb) and capsid protein (CP; ~1.4 kb), respectively (47). The RdRp-encoded genomic RNAs of EsNV1 (3,087 nt) and EsNV2 (3,018 nt) were found, but the genomic RNAs encoding CP were missing. The RdRp-encoded genomic RNAs of EsNV1 and EsNV2 both had a genome with one ORF encoding a methyltransferase domain and RdRp domain (Fig. 4).

Phylogenetic analysis revealed that EsNV1, Nodamura virus (NoV), and Shuangao insect virus 11 (SIV11) clustered together and fell within the genus *Alphanodavirus* (Fig. S6). The RdRp-encoded sequence of EsNV1 shared 58.1% amino acid identity with NoV and 66.2% with SIV11. Based on the ICTV report, definitive species demarcation within the genus *Alphanodavirus* requires the nucleotide sequence of the viral CP gene, as the two closest recognized species in this genus *Black beetle virus* and *Flock house virus* share 81% to 87% amino acid sequence identity in CP but are 99% identical in RdRp (47). The limited similarity of RdRp between EsClV and SIV11 suggests that EsNV1 may be considered a novel viral species in *Alphanodavirus.* The genomic RNA encoding CP of EsNV1 needs to be discovered and used to definitively classify EsNV1 in the future.

EsNV2 formed a clade with Yunnan sediment noda-like virus 1 that is sister to current two genera of the family *Nodaviridae* in the phylogenetic tree (Fig. S6). EsNV2 shares limited similarity (47.8% amino acid identity) with Yunnan sediment noda-like virus 1, indicating that it was a novel virus and may represent a novel genus in *Nodaviridae*. The establishment of this novel genus requires more characteristics of EsNV2 and similar viruses, including the genomic RNAs encoding CP and other biological properties.

EsNlV, Wenzhou crab virus 4, and Wenzhou tombus-like virus 18 formed a clade that is sister to the family *Sinhaliviridae*, based on the phylogenetic tree (Fig. S6). EsNlV shared 59.4% amino acid identity with Wenzhou crab virus 4 and 59.6% amino acid identity with Wenzhou tombus-like virus 18, suggesting EsNlV should be considered a new virus. The genome of EsNlV is 4,802 bp in length and has three ORFs that contain a methyltransferase domain, RdRp domain, and coat protein subunit, respectively (Fig. 4). Only two viruses that infect the western honeybee are currently classified as members of *Sinhaliviridae*, and these have non-segmented genomes that are ~5,900 nt in length and encode three ORFs (48). Unlike typical genomes of the family *Nodaviridae*, the genomes of EsNlV and its similar viruses are monopartite and encode three ORFs, showing more similarity to *Sinhaliviridae* than to *Nodaviridae*. The assignment of EsNlV requires further research in the future.

**(v) Viruses in the order *Tolivirales*.** *Tombusviridae* is a large family of viruses that infect monocotyledonous or dicotyledonous plants (49). Three subfamilies of *Tombusviridae* were created in 2018 based on their RdRp expression mechanisms and general gene order (50). Although *Tombusviridae* are considered plant viruses, previous studies have discovered a number of viruses closely related to members of *Tombusviridae* in crustaceans (4). Here, we also found two viruses closely related to the *Tombusviridae* subfamily *Procedovirinae* in Chinese mitten crabs. We identified a virus previously reported as Tombusviridae sp. isolate 258-k141_72490 (1,165 nt, GenBank accession no. ON049950) that contained a partial RdRp domain, and renamed it Eriocheir sinensis tombusvirus 1 (EsTV1). We generated a nearly complete genome of EsTV1 of 4,861 nt in length that contained P33 protein, RdRp domain, and a coat protein subunit (Fig. 4). Additionally, a novel tombusvirus with a length of 4,283 nt was identified and named Eriocheir sinensis tombusvirus 2 (EsTV2). EsTV2 has a similar genome organization to that of EsTV1, except that the function of its 5′ ORF is unknown. The genome organizations of EsTV1 and EsTV2 were similar to those of the subfamily *Procedovirinae*.

Phylogenetic analysis showed that EsTV1, EsTV2, and their related viruses formed a clade sister to the genus *Avenavirus* in the subfamily *Procedovirinae* (Fig. S7). EsTV2 shared limited similarity with Sanya tombus-like virus 2 (58.2% amino acid identity) and Forsythia suspensa tombusvirus (55.6% amino acid identity), suggesting that EsTV2 is a novel virus. The similarity of genome organization between EsTVs and the subfamily *Procedovirinae* and the phylogeny of EsTVs indicated that these viruses may represent novel genera of *Procedovirinae* in the family *Tombusviridae*. The phylogenetic result also showed that *Procedovirinae* was paraphyletic due to the introduction of EsTV1, EsTV2 and their relatives (Fig. S7). The phylogeny of *Procedovirinae* needs to be confirmed using more reference sequences in the future.

**(vi) Viruses in the order *Timlovirales*.** The taxonomy of positive single-stranded RNA phages (+ssRNA phages) has been restructured due to the rapidly increasing number of known +ssRNA phages (51, 52). In 2021, the class *Allassoviricetes* was renamed *Leviviricetes* and greatly expanded, resulting in an enormous taxonomic structure of 2 orders, 6 families, 428 genera, and 882 species. Two new +ssRNA phages belonging to the family *Blumeviridae*, order *Timlovirales*, are reported here. Eriocheir sinensis blumevirus 1 (EsBLV1) with 5,266 nt and Eriocheir sinensis blumevirus 2 (EsBLV2) with 3,514 nt both had genomes containing 3 ORFs: their 3′ ORF encoded the RdRp, while the functions of the other ORFs were unknown (Fig. 4). Based on the phylogenetic tree constructed using amino acid sequences encoding the RdRp, EsBLV1, EsBLV2, Beihai levi-like virus 12 (BLlV12), and two previously reported +ssRNA phages formed a clade together, but their branches were all long, meaning that these viruses were genetically distant from each other (Fig. S8). EsBLV1 shared 40.91% RdRp identity with Riboviria sp. isolate goldenpheasant222con20 (GenBank accession no. MW239254), but they did not cluster together in the phylogenetic tree. The criterion for establishing a novel genus in the class *Leviviricetes* is 50% pairwise amino acid identity of the RdRp (52). EsBLV1, EsBLV2, BLlV12, and their closely related viruses shared low amino acid identity of the RdRp with each other (34.4% to 40.9%), indicating that EsBLV1 and EsBLV2 represented novel genera in the family *Blumeviridae*. Here, we propose to establish two novel genera for EsBLV1 and EsBLV2, with the genus containing EsBLV1 named “Craspvirus,” standing for crab ssRNA phage, and the genus containing EsBLV2 named “Braspvirus,” standing for brachyuran ssRNA phage.

**(vii) Viruses in the order *Ourlivirales*.** The family *Botourmiaviridae* in the order *Ourlivirales* includes viruses infecting plants and fungi; most of its genera, except for *Ourmiavirus* (tripartite), have a non-segmented genome (2.0 to 5.3 kb) containing one ORF that encodes the RdRp (53). Two novel viral sequences related to the family *Botourmiaviridae* were identified in this study. Eriocheir sinensis botourmia-like virus 1 (EsBOlV1) with a length of 3,493 nt and Eriocheir sinensis botourmia-like virus 2 (EsBOlV2) with a length of 3,404 nt both had a genome containing only one ORF which encoded the RdRp (Fig. 4). EsBOlV1 and EsBOlV2 were most similar to each other, showing 88.0% amino acid identity in a genome-wide scale. Phylogenetic results showed that EsBOlV1, EsBOlV2, and their similar viruses cluster together and are relatively close to the genus *Ourmiavirus* of *Botourmiaviridae* (Fig. S9), but the genome organizations of these viruses were more similar to those in other *Botourmiaviridae* genera than to those in *Ourmiavirus*. The distinct hosts of EsBOlVs and the long branch length of the clade containing EsBOlVs indicated that EsBOlV1 and EsBOlV2 may represent a novel genus in the family *Botourmiaviridae*. Here, we propose to establish a novel genus named “Crabovirus” for EsBOlVs. This name was derived from the crabs’ botourmia-like virus.

**(viii) Viruses in the order *Picornavirales*.** *Picornavirales* is a large order of positive-sense single-stranded RNA viruses and currently encompasses 8 families, 104 genera, and 344 species (54). Here, we found 11 viruses related to 4 families in the order *Picornavirales*.

*Picornaviridae* is the largest family in *Picornavirales* and encompasses a number of viruses that infect vertebrates (55). Previous studies divided members of *Picornaviridae* into 5 subfamilies; in addition to the viruses assigned to these 5 subfamilies, *Ampivirus A1*, a species detected in newt feces (Lissotriton vulgaris), showed high divergence and formed a clade far away from all other genera in the phylogenetic tree (56). The limited availability of sequences for *Ampivirus* prevented further taxonomic assignment to a current or new subfamily. In this study, we found two viruses closely related to the species *Ampivirus A1* in Chinese mitten crabs. A novel picorna-like virus sequence with a length of 8,297 bp, named Eriocheir sinensis picorna-like virus (EsPlV), was discovered. A previously reported virus, Shahe picorna-like virus 13 (SPlV13), with a length of 9,077 bp, was also found. These two viruses both had typical genome organizations of *Picornaviridae*: a long ORF containing a coat protein near the 5′ untranslated region (UTR) and a helicase, protease, and RdRp replication module (Hel-Pro-RdRp) near the 3′ UTR (Fig. 4). Phylogenetic results showed that EsPlV, SPlV13, Ampivirus sp. isolate 180b-k141_71509, and *Ampivirus A1* were clustered as a clade distant from other *Picornaviridae* subfamilies (Fig. S10). Our phylogenetic tree also showed that the subfamily *Paavivirinae* of *Picornaviridae* clustered with the family *Caliciviridae* instead of with other subfamilies, indicating that the family *Picornaviridae* might be a paraphyletic group.

*Dicistroviridae* is a family of RNA viruses with genomes containing two ORFs encoding nonstructural proteins (Hel-Pro-RdRp module) near the 5′ UTR and capsid proteins near the 3′ UTR, respectively (57). Three novel virus sequences and a reported virus discovered in our work were related to the family *Dicistroviridae*. A novel virus sequence, named Eriocheir sinensis dicistro-like virus (EsDlV), was 9,308 nt long. EsDlV shared 70.2% amino acid identity with Shahe arthropod virus 1 (SAV1, 9,289 nt), which was also found in our study. EsDlV and SAV1 both had a typical genome organization of the family *Dicistroviridae* (Fig. 4). The phylogenetic tree showed that EsDlV and SAV1 clustered together and formed a clade sister to a big clade containing the families *Dicistroviridae*, *Secoviridae*, *Iflaviridae*, and *Marnaviridae* with low confidence (Fig. S10). The clade containing EsDlV and SAV1 was closer to *Dicistroviridae* than to other families. Due to the similar genome organization to *Dicistroviridae* and the phylogenetic results, we suggest that EsDlV and SAV1 may represent a new group of the family *Dicistroviridae*.

Two novel virus sequences were located within the family *Dicistroviridae* (Fig. S10). Eriocheir sinensis dicistrovirus 1 (EsDV1), with a length of 7,577 nt, clustered with Changjiang picorna-like virus 11 and Riboviria sp. isolate 5cz-RDRP-2 and formed a clade that was sister to the genus *Triatovirus*. Eriocheir sinensis dicistrovirus 2 (EsDV2), with a length of 7,116 bp, clustered with Wenzhou shrimp virus 5 and Wenzhou shrimp virus 4 and formed a clade that was sister to the members of the genus *Aparavirus* isolated from crustaceans. According to the ICTV report, the species demarcation criterion for the family *Dicistroviridae* is 90% amino acid identity of the CP (57). EsDV1 and EsDV2 shared less than 38% amino acid identity of the CP with their most similar viruses, suggesting that they are novel viral species in *Dicistroviridae*. Notably, our phylogenetic results showed that the genera *Aparavirus* and *Cripavirus* were both paraphyletic groups, suggesting that the current assignment of genera was incompatible with the expanding members of *Dicistroviridae*.

*Iflaviridae* is a family of viruses with RNA genomes approximately 9 to 11 kb in length that encode a single polyprotein (58). All members of this family have been isolated from arthropods. A novel sequence with partial genome encoding protease and RdRp, named Eriocheir sinensis iflavirus 1 (EsIV1), was found in this study (Fig. 4). In the phylogenetic tree, EsIV1 clustered with members of the family *Iflaviridae*, suggesting that EsIV1 was a possible new member of *Iflaviridae* (Fig. S10). The species demarcation criteria of *Iflaviridae* is 90% amino acid identity of the capsid proteins (58). The establishment of a new species in the family *Iflaviridae* requires the discovery of the complete EsIV1 genome in the future.

*Marnaviridae* is a greatly expanding family of viruses with RNA genomes 8.6 to 9.6 kb in length that encode one or two ORFs (59). Some members of *Marnaviridae* have been found in marine protists, and some are known from metagenomic studies of marine and freshwater environments and aquatic invertebrates (4, 59). Four new marnavirus sequences were identified in this study, including Eriocheir sinensis marnavirus 1 (EsMV1, 8,832 nt, 2 ORFs), Eriocheir sinensis marnavirus 2 (EsMV2, 8,634 nt, 2 ORFs), Eriocheir sinensis marnavirus 3 (EsMV3, 8,142 nt, 1 ORF), and Eriocheir sinensis marnavirus 4 (EsMV4, 7,809 nt, 2 ORFs). Phylogenetic analysis showed that EsMV1, EsMV2, EsMV3, and the members of the genus *Locarnavirus* formed a clade (Fig. S10). EsMV4 appears sister to this clade, suggesting that these marnaviruses were potential members of *Locarnavirus*. The species demarcation criteria of the genus *Locarnavirus* are based on pairwise comparisons of the RdRP (90% identity) and capsid (75%) amino acid sequences (59). EsMV1 shared 89.3% amino acid identity of RdRp and 98.4% amino acid identity of capsid with Sanya marna-like virus 2 (SMlV2), indicating that EsMV1 and SMlV2 may represent one novel species in the genus *Locarnavirus*. EsMV2 shared 65.3% amino acid identity of RdRp and 49.0% amino acid identity of capsid with Beihai picorna-like virus 47 (BPlV47), suggesting that EsMV2 may belong to a novel species in *Locarnavirus*. EsMV3 shared 40.8% amino acid identity of RdRp and 47.5% amino acid identity of capsid with Picornavirales sp. isolate HPLV-22 (GenBank accession no. OM622277), indicating that EsMV3 may also represent a novel species in *Locarnavirus*. EsMV4 shared 41.6% amino acid identity of RdRp sequence with mute swan feces-associated picorna-like virus 18 and shared <35.5% amino acid identity of capsid sequence with all similar viral sequences in the NCBI nonredundant protein (nr) database, suggesting that EsMV4 may also belong to a novel species.

**(ix) Virus in the order *Sobelivirales*.** There are three families in the order *Sobelivirales*: *Alvernaviridae*, *Barnaviridae*, and *Solemoviridae*. Members of these families possess genomes longer than 4,000 nt (60–62). Two sobemo-like virus sequences were found in this study. Eriocheir sinensis sobemo-like virus 1 (EsSlV1), with a length of 3,124 bp, and Eriocheir sinensis sobemo-like virus 2 (EsSlV2), with a length of 3,046 bp, have similar genome structures that contain a longer ORF encoding peptidase S1 and a shorter ORF encoding RdRp (Fig. 3). Compared to current members of *Sobelivirales*, EsSlV1 and EsSlV2 have shorter genomes and lack the ORFs encoding capsid proteins. EsSlV1 shared low amino acid identity with Norway luteo-like virus 3 (38.2%) and Ixodes scapularis-associated virus 1 (38.7%). EsSlV2 shared 88.6% amino acid identity with Beihai sobemo-like virus 24. EsSlV1, EsSlV2, and their similar viruses formed a distinct clade and were distantly related to the current families of *Sobelivirales* in the phylogenetic tree constructed using the RdRp, indicating that EsSlVs may represent a novel group at the family level in *Sobelivirales* (Fig. S11). We propose to establish a novel family for EsSlVs, named “Arthsoviridae,” which is derived from arthropod sobemo-like viruses.

## DISCUSSION

Many viromic studies have employed multi-location sampling to increase viral diversity and draw reliable conclusions. In our work, we observed high regional variation in viral species composition, with most of the viral species being region-specific, indicating the importance of multi-location sampling. For viromic research focused on uncovering novel viruses and expanding viral diversity, variation in viral species composition is an important component of viral diversity and should not be ignored. For viromic research focused on pathogen investigation, multi-location sampling can increase the reliability of analysis and exclude misleading regional differences.

We speculate that milky disease and hepatopancreatic necrosis syndrome are not mainly caused by viruses. The pathogen of MD was found to be the yeast Metschnikowia bicuspidate (24). Thirteen viral species which had higher relative abundances in MD-affected crab sample may have synergistic effects on the development of MD. A previous study reported that microsporidia were detected in the hepatopancreas of HPNS-affected crabs (25), and environmental stress, nutrition, and toxicants are also considered probable causes of HPNS (26, 63). The pathogen of HPNS is still unclear. Based on the comparison of relative abundance in the samples from Dongying and Suqian, none of the viral species were identified as potential pathogens of HPNS, suggesting that viruses are not the main cause of HPNS. The comparison of viromes between diseased crabs and asymptomatic crabs could provide new clues as to whether viruses play a role in the outbreak of diseases.

In current virus taxonomy, members from the same family or genus generally share similar host preferences. However, with the discovery of a large number of new viruses, many viruses found in organisms from different phyla and even different kingdoms show close evolutionary relationships (4). We noticed that a few viruses found in our study showed close relationships with plant viruses, especially as some of them exhibited higher abundance in diseased crabs than in asymptomatic crabs. EsKlV occupied a phylogenetic position within the family *Kitaviridae*, which currently consists of plant viruses, and it was highly abundant in diseased crabs from Tangshan and Suqian. EsBOlV1 was slightly more abundant in all libraries of diseased crabs than in those of asymptomatic crabs and clustered into a family consisting of plant and fungi viruses. EsTV2 (novel virus) shared the highest similarity with two viruses from two traditional Chinese herbs, Leptochloa chinensis and Forsythia suspensa. A previous study reported that Macrobrachium rosenbergii virus 15, found in the giant freshwater prawn Macrobrachium rosenbergii, shared >99% amino acid identity with two viruses found in two traditional Chinese herbs, Trichosanthes kirilowii and F. suspensa (19). An important pathogen of the giant freshwater prawn, Macrobrachium rosenbergii nodavirus was found to have surprising similarities in its capsid structure with members of the family *Tombusviridae* which infect plants (64). The similarities between viruses found in crustaceans and those found in plants indicate the possibility of cross-species transmission. The transmission of viruses may introduce the risk of new diseases and harm the cultivation of crustaceans. The potential risk of cross-species transmission deserves to be systematically investigated in the future. In addition, the gills of crabs were pooled with other tissues for library construction, so the viruses that showed close relationships with plant viruses in our study may have come from the aquatic environment or from other organisms.

In summary, our findings showed that the Chinese mitten crab *E. sinensis* harbors many different viruses, most of which were first reported here. Four viral orders were found to be potential threats to the cultivation of Chinese mitten crabs based on their relative abundance between diseased and asymptomatic crabs. Furthermore, the viruses were classified based on their phylogenetic results, genome structures, and host preferences. We propose the establishment of three novel families and five novel genera in total. Our knowledge of viromes in Chinese mitten crab provides a foundation for further study of brachyuran crustacean aquaculture and virus taxonomy in the future.

## MATERIALS AND METHODS

To explore the differences in viral composition among samples, we approached the analysis from several perspectives, including the number of viral species shared among different samples, the relative abundance of viral species in each sample, and the relative abundance of viral orders in each sample. Clean reads were mapped to 31 viral genomes and the number of reads or fragments that aligned to each viral genome was counted. For viral species with genomes of different lengths, we use fragments per kilobase of exon per million fragments mapped (FPKM) as the relative abundance. To assess the number of viral species in the sample and avoid false positive alignment, a species for which the number of mapped reads per million total reads (RPM) was greater than 10 was considered to be present in the sample. For higher viral taxa, we used RPM as the relative abundance because it is difficult to normalize the total amount of reads by length for multiple sequences from different viruses.

### Sample collection.

Chinese mitten crabs with or without diseases were collected from farming ponds in three different regions in China (Table 1). Specifically, 10 crabs with MD and 10 asymptomatic crabs were collected in Tangshan, Hebei Province in April 2021; 10 HPNS-affected crabs and 10 asymptomatic crabs were collected in Dongying, Shandong Province in August 2021; and finally, 10 HPNS-affected crabs and 10 asymptomatic crabs were collected in Suqian, Jiangsu Province in October 2021. The hepatopancreas, branchia, and muscle tissues of each crab were harvested and stored in RNAlater (Invitrogen), then transported to Sun Yat-sen University on dry ice and stored at −80°C until use. We strictly complied with biosafety regulations in all of our experiments.

### Sequencing library construction and sequencing.

The hepatopancreas, branchia, and muscle tissues of 10 diseased or 10 healthy crabs collected from each region were pooled, resulting in 6 pools for sequencing library construction. The sample pools were homogenized with phosphate-buffered saline (PBS) (10% [mass/vol]) in a Lu kα sample freezing grinder for 3 min at 60 Hz and 4°C, frozen, and thawed three times on dry ice. The suspension of each pool was subjected to centrifugation for 10 min at 12,000 × *g*. The supernatant was collected and filtered through a Durapore PVDF (polyvinylidene difluoride) 0.45-μm-pore filter (Millipore) to remove eukaryotic and bacterial cell-sized particles. The filtrates were subjected to ultracentrifugation (4°C, 180,000 × *g*) for 2 h to enrich the virus-like particles. After supernatant was carefully removed, the virus-like particles were resuspended for RNA extraction. RNA was extracted using a QIAamp Viral RNA minikit (Qiagen) in accordance with the manufacturer’s protocol. The quality and quantity of the RNA were measured on a Qubit 4.0 Fluorometer (Invitrogen) using the Qubit RNA HS assay kit.

The rRNA was removed for library construction by the NEBNext rRNA Depletion kit (New England Biolabs [NEB]). RNA sequencing libraries were constructed using a NEBNext Ultra II RNA Library Prep kit for Illumina (NEB) in accordance with the manufacturer’s protocol. The concentration of libraries was measured on a Qubit 4 Fluorometer (Invitrogen) using the Qubit dsDNA HS assay kit. Sequencing for the pair-end libraries were performed with 2 × 150 bp chemistry on the Illumina NovaSeq 6000 platform (Illumina).

### Sequence assembly and virus discovery.

For each library, sequencing reads were first quality controlled using fastp (version 0.21.0) (65) and assembled *de novo* using SPAdes (version 3.15.3) (66) in rnaviral mode. Assembled contigs longer than 500 bp remained and were clustered using CD-HIT (version 4.8.1) (67). Contigs were then compared against the NCBI nr database using Diamond blastx (version 2.0.6) (68). Taxonomic lineage information was obtained for the top blast hit of each contig and reformatted using TaxonKit (version 0.7.2) (69), and contigs belonging to the kingdom “Viruses” were identified as probable virus hits. Viral contigs with unassembled overlaps were merged using the SeqMan program implemented in the Lasergene software package (version 7.1.0) (70).

To confirm the assembly results and limit false positives, quality-controlled reads were mapped to viral contigs using Bowtie2 (version 2.4.2) (71) and SAMtools (version 0.1.19) (72), and contigs for which the number of mapped reads per million total reads (RPM) in a library was greater than 10 were used for follow-up analysis.

### Viral genome annotation.

Viral contigs were manually assigned to specific virus species by comparing their sequence similarity to each other and to the most similar sequences from nr database, based on the species demarcation criteria of specific taxa from the ICTV Report Chapters by Genome (https://ictv.global/report/genome). The ORFs of viral genomes were identified using ORFfinder (73). Protein function prediction was performed using InterProScan (version v5.52-86.2) (74). Genome organizations were visualized with ChiPlot (https://www.chiplot.online/). The novel viruses found in this study were named according to the scientific name of the Chinese mitten crab and the phylogenetic analysis described below.

### Phylogenetic analysis and virus abundance analysis.

To determine the evolutionary relationships between viruses detected in this study and ICTV-designated species, we used the amino acid sequences of RdRp to construct phylogenetic trees. The amino acid sequences of RdRp were obtained by comparing the amino acid sequences of ORFs to the RdRp-scan database (version 0.90) (75) using Diamond blastp (version 2.0.6). The viral replicase sequence alignments were performed by mafft (version 7.310) (76) employing the L-INS-i algorithm, then trimmed using trimAL (77) with the default parameters encapsulated in TBtools (version 1.098669) (78). Phylogenetic trees were generated using IQ-TREE (version 1.6.12) (79) with the maximum-likelihood method. Branch support was estimated using both the SH-like approximate likelihood ratio test (SH-aLRT) and ultrafast bootstrap approximation (UFBoot) (80). Tree files were visualized with Interactive Tree of Life (iTOL, version 6) (81).

To access the relative abundances of virus species in each library, quality-controlled reads were mapped to the whole genome of virus species using Bowtie2, and the FPKM of each virus species in each library was calculated using featureCounts (version 2.0.2) (82). The heatmap was drawn in RStudio (version 1.3.959) using the pheatmap package (version 1.0.12).

### Confirmation of viral genomes.

To verify the presence of the RdRp genes, we used the original samples for RT-PCR and nested RT-PCR with primer sets designed based on the RdRp sequences of viral genomes. The PCR products were sequenced using Sanger sequencing and then compared with the assembled sequences. To calculate the genome coverage of reads and the heterozygosity of SNPs, raw sequencing reads were aligned to the assembled genomes using bwa (version 0.7.17) (83). Genome coverage was evaluated using CollectWgsMetrics function implemented in Picard (version 3.0.0). SNPs were identified using BCFtools (version 1.9) (84), and the heterozygosity of SNPs was then calculated for each assembled genome.

### Data availability.

Raw sequencing reads are accessible in NCBI under BioProject no. PRJNA921821. All viral sequences identified for the first time in this study have been deposited in GenBank under accession numbers OP019089 to OP019093, OP019095 to OP019108, and OP019111 to OP019132.
