# Supplementary material for: Virome Analysis Provides an Insight into the Viral Community of Chinese Mitten Crab Eriocheir sinensis
Source: Microbiol Spectr. 2023 Jun 26;11(4):e01439-23. doi: 10.1128/spectrum.01439-23 (PMC10433957; doi:10.1128/spectrum.01439-23)
Supplement: Supplemental file 1 — Supplemental material. Download spectrum.01439-23-s0001.docx, DOCX file, 4.1 MB [file spectrum.01439-23-s0001.docx]

**Supplementary Materials for**

**Virome analysis provides an insight into the viral community of Chinese mitten crab *Eriocheir sinensis***

**Supplementary Table 1**. The nested primers for amplifying and sequencing viruses identified in this study. The primers were designed based on the regions that encode the RdRp domain.

| **Virus name** | **Primer type** | **Forward** | **Reverse** | **Length (bp)** |
| --- | --- | --- | --- | --- |
| Eriocheir sinensis reovirus 1 | Outer primers | GGACAAGAGAACCAGGCTATTAC | CCAACCACCTCATTCATACCTAA | 684 |
|  | Inner primers | CAGCACATTCCTGTCTAAGTCTAT | CCAACCACCTCATTCATACCTAA | 321 |
| Eriocheir sinensis reovirus 2 | Outer primers | CCGCTCCTCTTCTCATTGATTC | TTGGTTGTTGTGGCTTGTGAA | 669 |
|  | Inner primers | CTCTTCTCATTGATTCAGCAGATG | CGTAGATGGCGATAGGTGTATG | 466 |
| Eriocheir sinensis reovirus 3 | Outer primers | GCTTTAGATGTCTCTGCCTTTG | CACTACCATACCTTCCAATCATTC | 547 |
|  | Inner primers | TCATACTACAACACGGCAACAG | CTACCATACCTTCCAATCATTCCTT | 337 |
| Chinese mitten crab virus 1 | Outer primers | GATACCAACATTGACATCCACTAAG | CAAGACAACAACCTTCATTCTGTAG | 742 |
|  | Inner primers | GATACCAACATTGACATCCACTAAG | TCTCTCTATCATCACCAGTCCTTT | 386 |
| Eriocheir sinensis bunyavirus | Outer primers | ACTCATAACGCAATGGGATTTC | ACAGCAGTGTTCATGTAGTACA | 584 |
|  | Inner primers | GTGGACTCAAGAATGGAAGGTATG | CAGCAGTGTTCATGTAGTACAGATT | 408 |
| Eriocheir sinensis alphatetra-like virus | Outer primers | AACTGTCAATCAGATCGCCATG | ATGTCACCAATCATTCTCGTCAG | 621 |
|  | Inner primers | GCAGACATCACAGAACAAGACA | CTCCCACCAGATACCCTATGAAA | 385 |
| Hubei sediment virgavirus 2 | Outer primers | TTAACTCGCCTGAATTGATGTCC | TATTGCCAATGAAGGTGGTGAC | 675 |
|  | Inner primers | TAACTCGCCTGAATTGATGTCC | ATACTGATGACCTCTACGCCAA | 586 |
| **Virus name** | **Primer type** | **Forward** | **Reverse** | **Length (nt)** |
| Eriocheir sinensis kita-like virus | Outer primers | CTAAGTTTGTCAATGCCGTCTTC | TCCTCTCATACTCTTCCGTCAG | 720 |
|  | Inner primers | GCTGGAGGTTGACATCTCTAAG | AGCAGAATAGGTGGTTGGAGTA | 401 |
| Eriocheir sinensis cholera-like virus | Outer primers | CAGGATTACCTCCAATGTTGTTG | TTGCGTTCCACTTCTGATGATT | 674 |
|  | Inner primers | CTAACCAATGAGATGTACGAGGAA | GTGACAATACAGTGCGTTGAGTA | 324 |
| Eriocheir sinensis nodavirus 1 | Outer primers | GCCGTACCGTAGACTTATTGAG | CAGGTTCTTAGTGGATCTTGGAA | 735 |
|  | Inner primers | CCGTAGACTTATTGAGTGCTTCC | GTTAAGATCACAGGTGGTTGGT | 454 |
| Eriocheir sinensis nodavirus 2 | Outer primers | GAAGAATTGTTGCGATGGACTG | TGTCTGGCTAAGAATTGAACTCC | 791 |
|  | Inner primers | GAAGAATTGTTGCGATGGACTG | TTGTTCACAGAGGTAGGCGATT | 324 |
| Eriocheir sinensis noda-like virus | Outer primers | CAAGGAGAACCAATGGCTACAG | CGTATGGCAAGTAATCGACTATGG | 711 |
|  | Inner primers | ACATCATCAACACCGTGGAGAA | CGTATGGCAAGTAATCGACTATGG | 321 |
| Eriocheir sinensis tombusvirus 1 | Outer primers | CGAGGCGTATGTTGGTTCTAAG | CATCACCGTTGTTCATCAGTTCT | 635 |
|  | Inner primers | AATGTTATGGAAGCAGGCAAGG | ATCACCGTTGTTCATCAGTTCTC | 363 |
| Eriocheir sinensis tombusvirus 2 | Outer primers | AACAAAGCCACGCACATACCAT | TTCGCATCTAATCCAATAGCAACTG | 606 |
|  | Inner primers | TTCAACAACAGTGTCTCGTCTG | AAGATACTTGCCAAGCGTCAAC | 384 |
| Eriocheir sinensis blumevirus 1 | Outer primers | CAACAAGGACTATTAGCTGCCATC | GAACTTACGGGAAATTCTTAACGGA | 600 |
|  | Inner primers | AACAAGGACTATTAGCTGCCATC | ATATGAATGTAACGGCGGAACC | 312 |

| **Virus name** | **Primer type** | **Forward** | **Reverse** | **Length (nt)** |
| --- | --- | --- | --- | --- |
| Eriocheir sinensis blumevirus 2 | Outer primers | GAACCATTACGACTGAGTCTACTG | CCACAGGATTCTCTGAATGTATCAT | 583 |
|  | Inner primers | CATTACGACTGAGTCTACTGGATAT | TCTTAGTTCTGGCAACGGATTC | 407 |
| Eriocheir sinensis botourmia-like virus 1 | Outer primers | CAAGTTGTACCTCTGTTAGAAGCAT | CCTCTTTCCATGTATCGTAATGTTC | 610 |
|  | Inner primers | CAAGTTGTACCTCTGTTAGAAGCAT | ACTGTATTTGAGTTGATGTCCTGTC | 369 |
| Eriocheir sinensis botourmia-like virus 2 | Outer primers | GAAGCATTCAAGGTTAGGACGAT | CCAGAGAGAATTTGAGACCACATT | 616 |
|  | Inner primers | AAGCATTCAAGGTTAGGACGAT | CCAAGTTAATCAGGCAGAGGAT | 426 |
| Eriocheir sinensis dicistrovirus 1 | Outer primers | TTGATCGCATCACCAACAAGAA | CCATAATGTCAGTCCACACCAA | 660 |
|  | Inner primers | TATGCTGGTGAGACTCGCCTTA | GGTAAACTCTTGTTCCACTGGTAAA | 395 |
| Eriocheir sinensis dicistrovirus 2 | Outer primers | GAGTTCTATCCTACGAAGAGGCTAT | CAGTGAGTTGATTATGACAGTGAGT | 716 |
|  | Inner primers | GCTGCTCCTCAAGATTATGTTATTC | CGAGTGCGTCCATTGTATGATAT | 348 |
| Eriocheir sinensis dicistro-like virus | Outer primers | GATTGGCAACAGATGGCAGATT | TATTGGGTCGTTCGTCTTGGTA | 636 |
|  | Inner primers | GATTGGCAACAGATGGCAGATT | AGTGAATGTCATACCCGCTTCT | 474 |
| Shahe arthropod virus 1 | Outer primers | GAACCAACAACAAGACCATCCA | CGAGTAATCTCCATCATTCACCAA | 657 |
|  | Inner primers | ACCATCCATTCTTCGTCCTTAC | AAGTGTTCCTCGTTCTGTTCTC | 548 |
| Eriocheir sinensis iflavirus 1 | Outer primers | GAGTCAAGTGGAGCATACTATCG | TCTCTGAGCGGCTTCTACATTA | 799 |
|  | Inner primers | TTGCGACTGGTATGAATACTACG | TCTCTGAGCGGCTTCTACATTA | 565 |
| **Virus name** | **Primer type** | **Forward** | **Reverse** | **Length (nt)** |
| Eriocheir sinensis marnavirus 1 | Outer primers | AGAACTCGTGGTGTTGTCAGAT | CCTTGAAGTCCTCGCAATCATC | 791 |
|  | Inner primers | GAAGACGAGCATATCCTGTGTAC | AGAGACTATTCACGAGCGAGTT | 510 |
| Eriocheir sinensis marnavirus 2 | Outer primers | TGGTAGAGCAACAGTTCACAGT | CATCTGAGCGTTCATCTTCTTGT | 629 |
|  | Inner primers | TGGTAGAGCAACAGTTCACAGT | TCTGAGGTTCGTCCTTCAAGTG | 372 |
| Eriocheir sinensis marnavirus 3 | Outer primers | TACTTGACAGAGCACGGTTATG | TGAATCTCAGCACTCACAACAG | 653 |
|  | Inner primers | GGAAGACAGGAGACAAGAAGTG | GGAATGCGTACCATCAACATCT | 508 |
| Eriocheir sinensis marnavirus 4 | Outer primers | ATCAGCAGGCTATGGACATACT | AGAATATGACCAGAAGGATTAGACC | 618 |
|  | Inner primers | GACGCCTGAATTAGTGGAAGAA | AGAATATGACCAGAAGGATTAGACC | 531 |
| Shahe picorna-like virus 13 | Outer primers | GGCAGTTAATGGTGTTGATGGA | ATCGTGGTAAATCCTCCTGGTA | 723 |
|  | Inner primers | ATTGCCATTGGAATCTGCCTTG | CGTGGTAAATCCTCCTGGTACT | 325 |
| Eriocheir sinensis picorna-like virus | Outer primers | AATAAGCATACAAGCGGCAAAC | TTGAGTTCACTAGCAACCTCTG | 800 |
|  | Inner primers | GAACTCGCAATAGAAATGGAAGG | CTCTGCTAGTAAGACTCGATCATAT | 432 |
| Eriocheir sinensis sobemo-like virus 1 | Outer primers | GCATCATATCAGCCGTATCTCTT | TCAAACTCAAATCCTGCGAACT | 616 |
|  | Inner primers | GCATCATATCAGCCGTATCTCTT | TGGAGCAAGGTATTAAGGACAATC | 421 |
| Eriocheir sinensis sobemo-like virus 2 | Outer primers | ATCTTGGAGAGTAGTCCTATGGTAG | GGCTGATGTCTGTTCTTCTGTC | 663 |
|  | Inner primers | CTTGGAGAGTAGTCCTATGGTAGT | TGCGGCTTCTGAATGGTATTAG | 324 |

**Supplementary Table 2.** Coverage of reads and heterozygosity of SNPs of assembled viral genomes.

| **Accession** | **Virus name** | **Genome length** | **Coverage of reads** | **Heterozygosity** |
| --- | --- | --- | --- | --- |
| OP019112- OP019124 | Eriocheir sinensis reovirus 1 | 20,173 | 99.65% | 0 |
| OP019125 | Eriocheir sinensis reovirus 2 | 4,486 | 100.00% | 0.09% |
| OP019095 | Eriocheir sinensis bunyavirus | 12,238 | 100.00% | 0.11% |
| OP019089 | Eriocheir sinensis alphatetra-like virus | 10,673 | 100.00% | 0 |
| OP019101 | Eriocheir sinensis kita-like virus | 12,933 | 100.00% | 0 |
| OP019096 | Eriocheir sinensis cholera-like virus | 18,830 | 100.00% | 0.38% |
| OP019107 | Eriocheir sinensis nodavirus 1 | 3,087 | 100.00% | 0 |
| OP019108 | Eriocheir sinensis nodavirus 2 | 3,018 | 100.00% | 0 |
| OP019106 | Eriocheir sinensis noda-like virus | 4,802 | 99.92% | 0 |
| OP019131 | Eriocheir sinensis tombusvirus 1 | 4,861 | 100.00% | 0.02% |
| OP019132 | Eriocheir sinensis tombusvirus 2 | 4,283 | 100.00% | 0 |
| OP019090 | Eriocheir sinensis blumevirus 1 | 5,266 | 99.85% | 0.13% |
| OP019091 | Eriocheir sinensis blumevirus 2 | 3,514 | 100.00% | 0 |
| OP019092 | Eriocheir sinensis botourmia-like virus 1 | 3,493 | 100.00% | 0 |
| OP019093 | Eriocheir sinensis botourmia-like virus 2 | 3,404 | 100.00% | 0 |
| OP019098 | Eriocheir sinensis dicistrovirus 1 | 7,577 | 100.00% | 0 |
| OP019099 | Eriocheir sinensis dicistrovirus 2 | 7,116 | 100.00% | 0.13% |
| OP019097 | Eriocheir sinensis dicistro-like virus | 9,308 | 100.00% | 0 |
| OP019100 | Eriocheir sinensis iflavirus 1 | 3,596 | 100.00% | 0 |
| OP019102 | Eriocheir sinensis marnavirus 1 | 8,832 | 100.00% | 0 |
| OP019103 | Eriocheir sinensis marnavirus 2 | 8,634 | 100.00% | 0 |
| OP019104 | Eriocheir sinensis marnavirus 3 | 8,142 | 100.00% | 0 |
| OP019105 | Eriocheir sinensis marnavirus 4 | 7,809 | 100.00% | 0 |
| OP019111 | Eriocheir sinensis picorna-like virus | 8,297 | 99.66% | 0.06% |
| OP019126 | Eriocheir sinensis sobemo-like virus 1 | 3,124 | 100.00% | 0.03% |
| OP019127 | Eriocheir sinensis sobemo-like virus 2 | 3,046 | 99.51% | 0.00% |


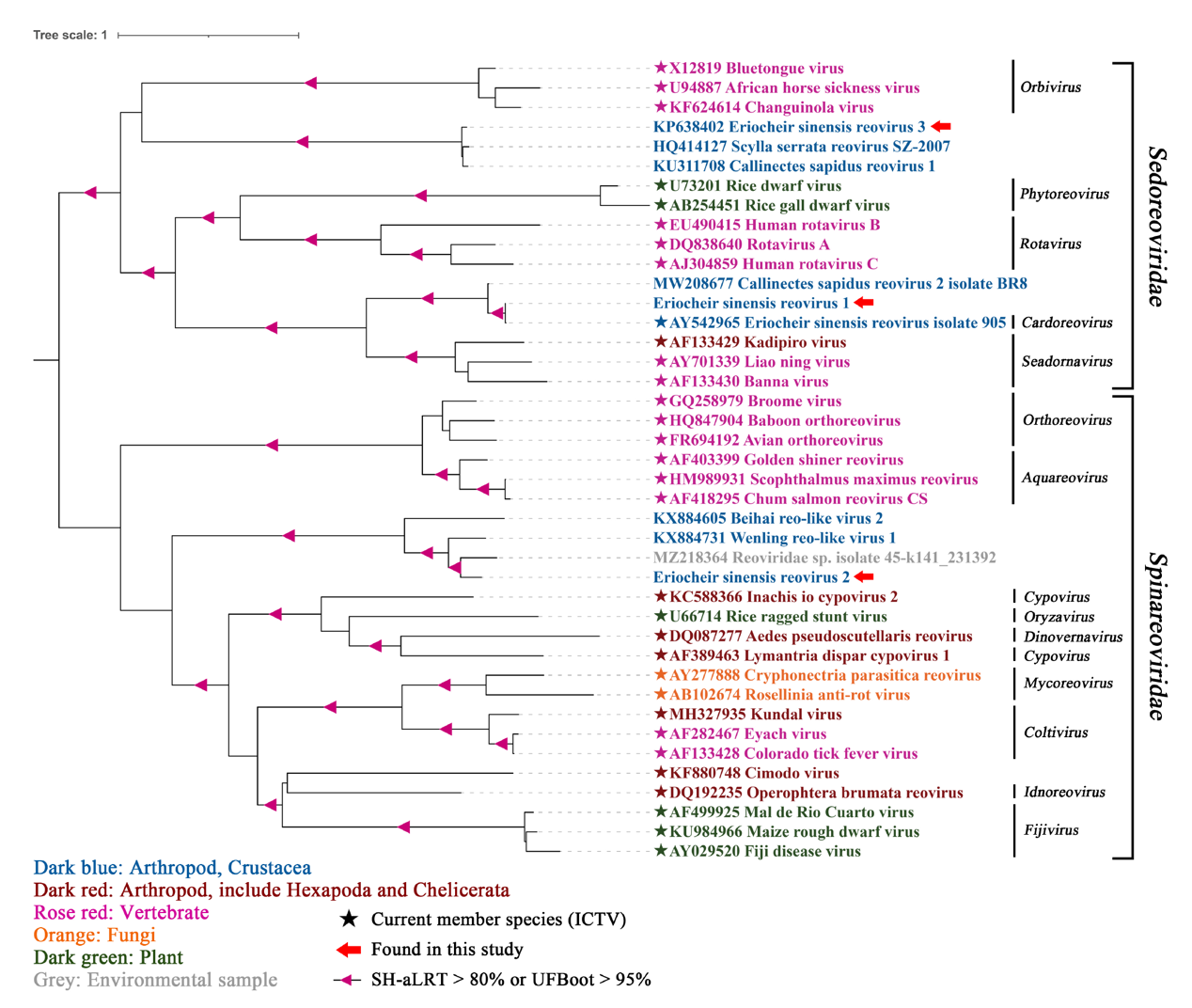


**Supplementary Figure 1. Phylogenetic tree of the order *Reovirales*.** A mid-point rooted maximum likelihood phylogenetic tree was constructed using IQ-TREE2 with amino acid sequences encoding RdRp. Branch supports were determined using the Shimodaira–Hasegawa approximate likelihood ratio test (SH-aLRT) and ultrafast bootstrap (UFBoot) approach with 1,000 replicates. Branches with high bootstrap supports are indicated by pink triangles (SH-aLRT > 80% or UFBoot > 95%). The hosts of viruses are denoted by different colors of virus names, and the ICTV-accepted members of families or genera are denoted by star symbols beside virus names. Viruses identified in this study are denoted by red arrows beside virus names.


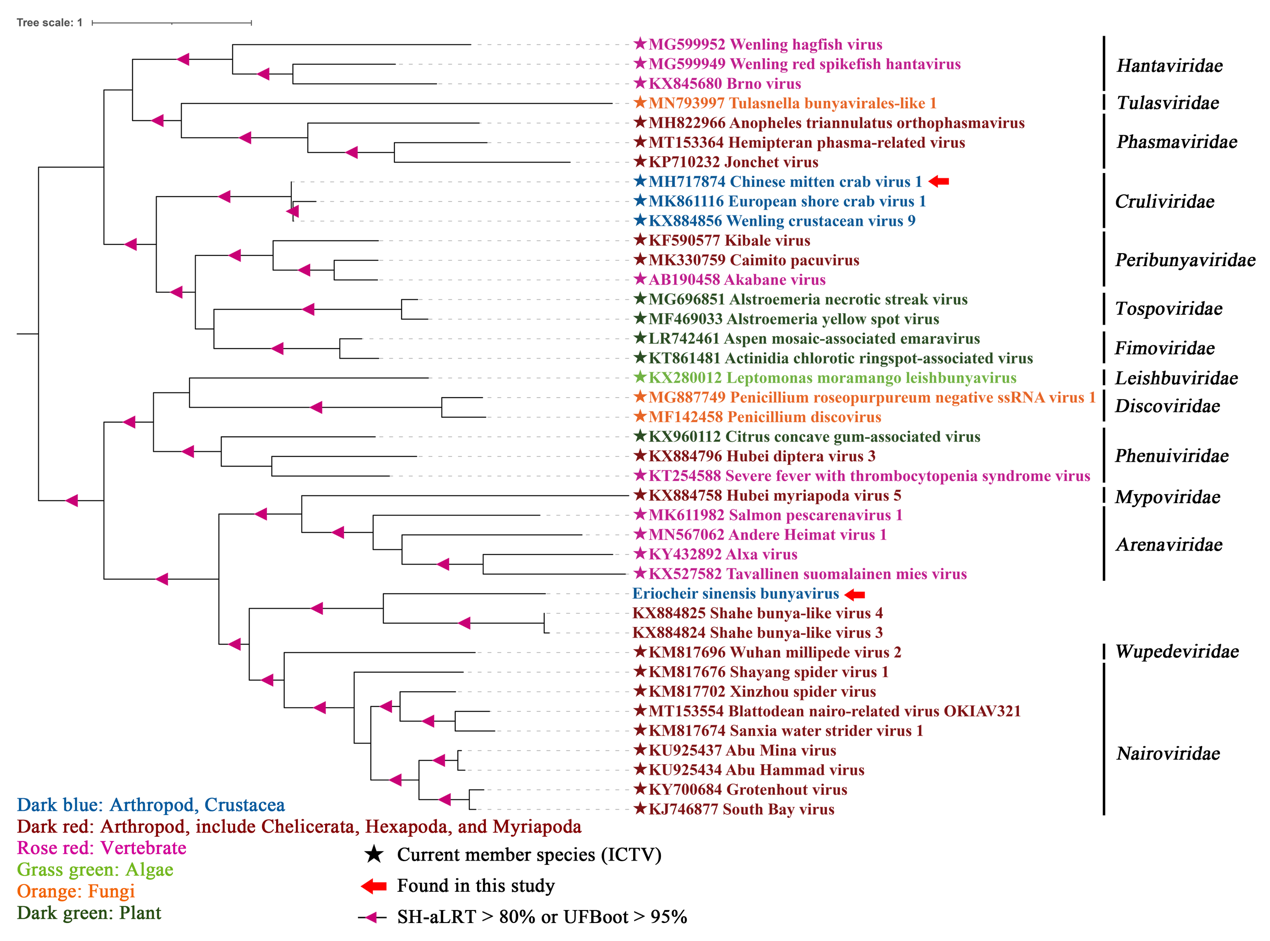


**Supplementary Figure 2. Phylogenetic tree of the order *Bunyavirales*.** A mid-point rooted maximum likelihood phylogenetic tree was constructed using IQ-TREE2 with amino acid sequences encoding RdRp. Branch supports were determined using the Shimodaira–Hasegawa approximate likelihood ratio test (SH-aLRT) and ultrafast bootstrap (UFBoot) approach with 1,000 replicates. Branches with high bootstrap supports are indicated by pink triangles (SH-aLRT > 80% or UFBoot > 95%). The hosts of viruses are denoted by different colors of virus names, and the ICTV-accepted members of families or genera are denoted by star symbols beside virus names. Viruses identified in this study are denoted by red arrows beside virus names.


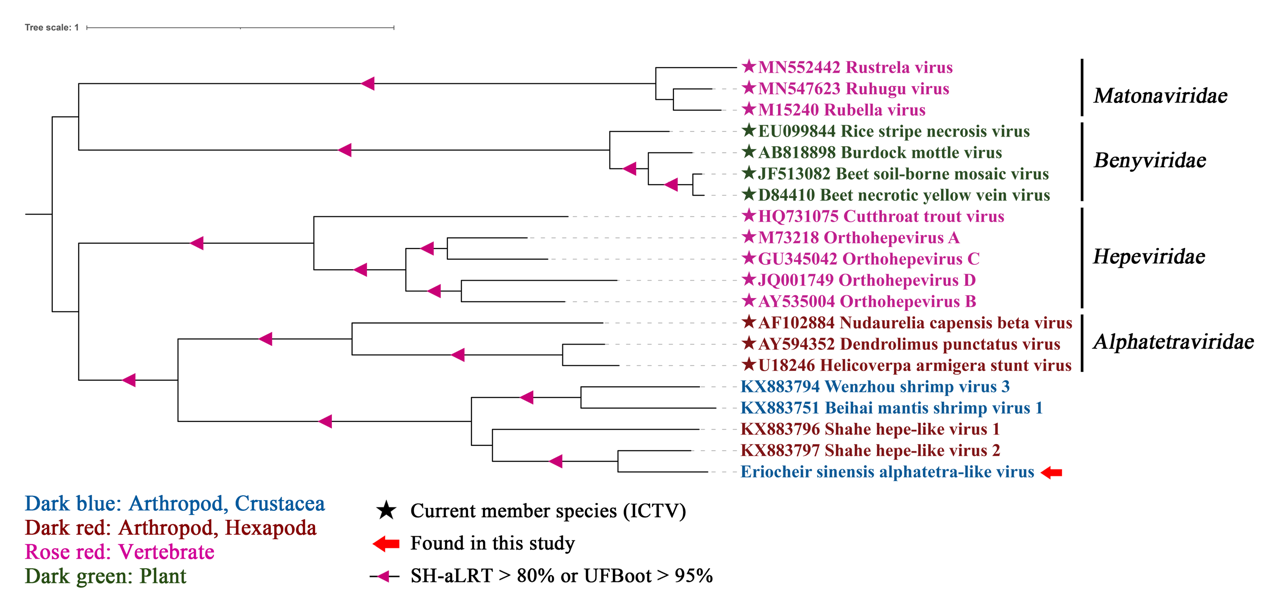


**Supplementary Figure 3. Phylogenetic tree of the order *Hepelivirales*.** A mid-point rooted maximum likelihood phylogenetic tree was constructed using IQ-TREE2 with amino acid sequences encoding RdRp. Branch supports were determined using the Shimodaira–Hasegawa approximate likelihood ratio test (SH-aLRT) and ultrafast bootstrap (UFBoot) approach with 1,000 replicates. Branches with high bootstrap supports are indicated by pink triangles (SH-aLRT > 80% or UFBoot > 95%). The hosts of viruses are denoted by different colors of virus names, and the ICTV-accepted members of families or genera are denoted by star symbols beside virus names. Virus identified in this study is denoted by red arrow beside virus name.


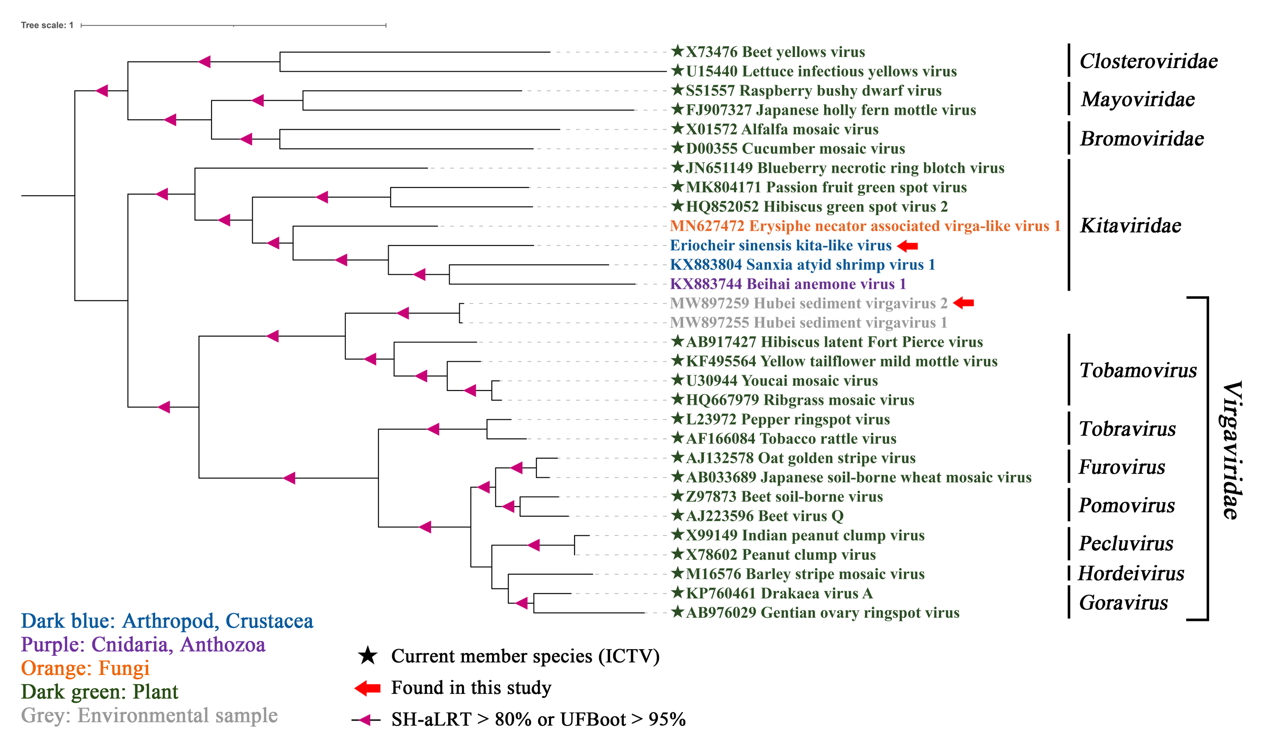


**Supplementary Figure 4. Phylogenetic tree of the order *Martellivirales*.** A mid-point rooted maximum likelihood phylogenetic tree was constructed using IQ-TREE2 with amino acid sequences encoding RdRp. Branch supports were determined using the Shimodaira–Hasegawa approximate likelihood ratio test (SH-aLRT) and ultrafast bootstrap (UFBoot) approach with 1,000 replicates. Branches with high bootstrap supports are indicated by pink triangles (SH-aLRT > 80% or UFBoot > 95%). The hosts of viruses are denoted by different colors of virus names, and the ICTV-accepted members of families or genera are denoted by star symbols beside virus names. Viruses identified in this study are denoted by red arrows beside virus names.


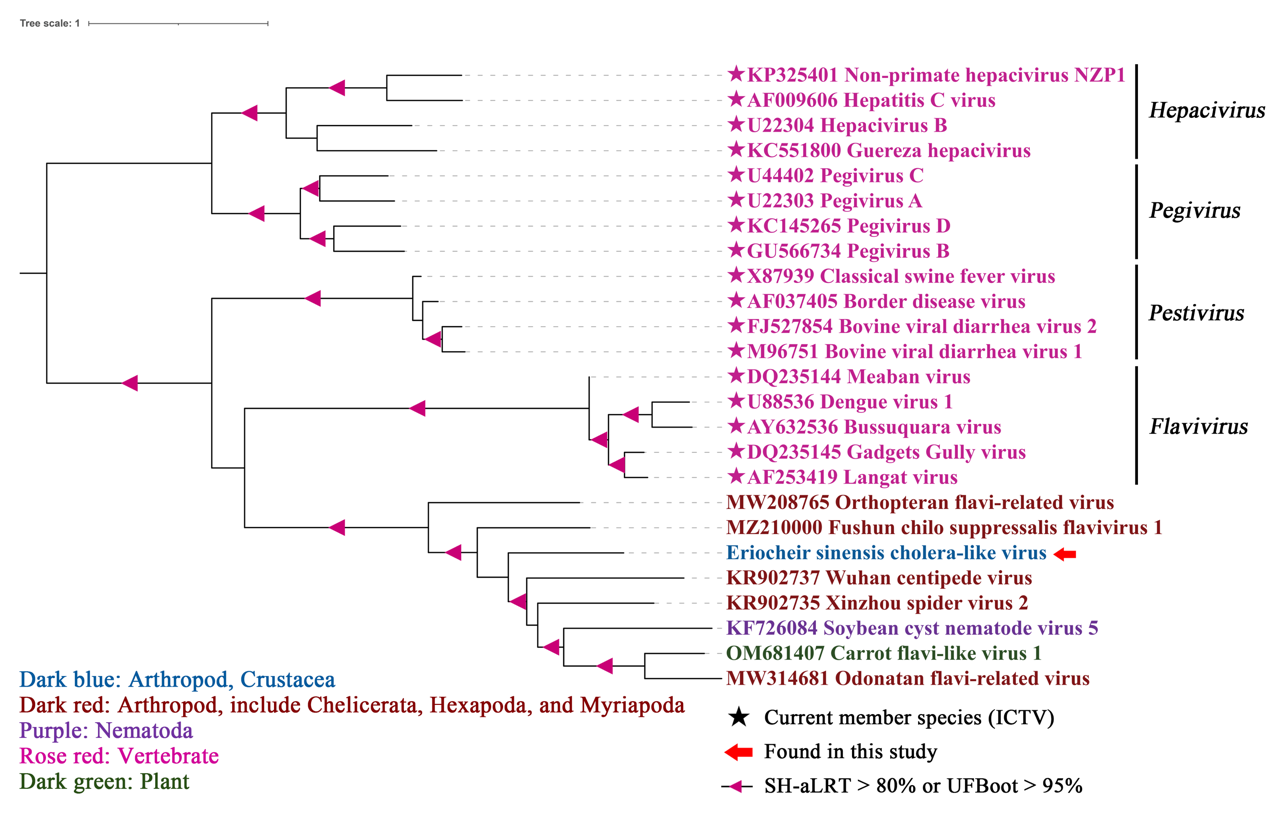


**Supplementary Figure 5. Phylogenetic tree of the order *Amarillovirales*.** A mid-point rooted maximum likelihood phylogenetic tree was constructed using IQ-TREE2 with amino acid sequences encoding RdRp. Branch supports were determined using the Shimodaira–Hasegawa approximate likelihood ratio test (SH-aLRT) and ultrafast bootstrap (UFBoot) approach with 1,000 replicates. Branches with high bootstrap supports are indicated by pink triangles (SH-aLRT > 80% or UFBoot > 95%). The hosts of viruses are denoted by different colors of virus names, and the ICTV-accepted members of families or genera are denoted by star symbols beside virus names. Virus identified in this study is denoted by red arrow beside virus name.


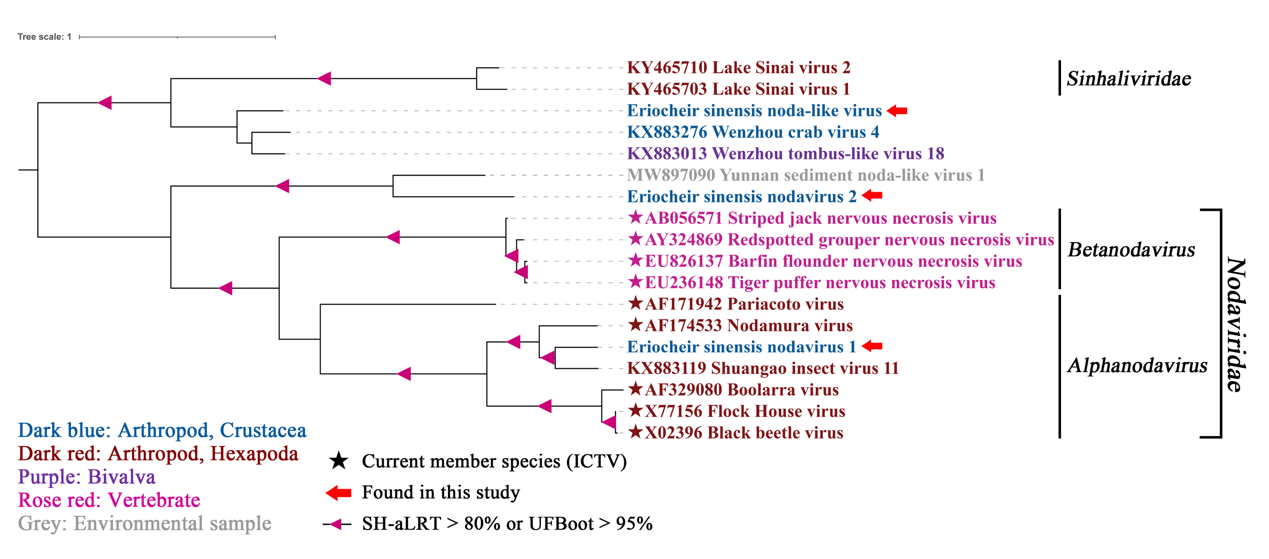


**Supplementary Figure 6. Phylogenetic tree of the order *Nodamuvirales*.** A mid-point rooted maximum likelihood phylogenetic tree was constructed using IQ-TREE2 with amino acid sequences encoding RdRp. Branch supports were determined using the Shimodaira–Hasegawa approximate likelihood ratio test (SH-aLRT) and ultrafast bootstrap (UFBoot) approach with 1,000 replicates. Branches with high bootstrap supports are indicated by pink triangles (SH-aLRT > 80% or UFBoot > 95%). The hosts of viruses are denoted by different colors of virus names, and the ICTV-accepted members of families or genera are denoted by star symbols beside virus names. Viruses identified in this study are denoted by red arrows beside virus names.


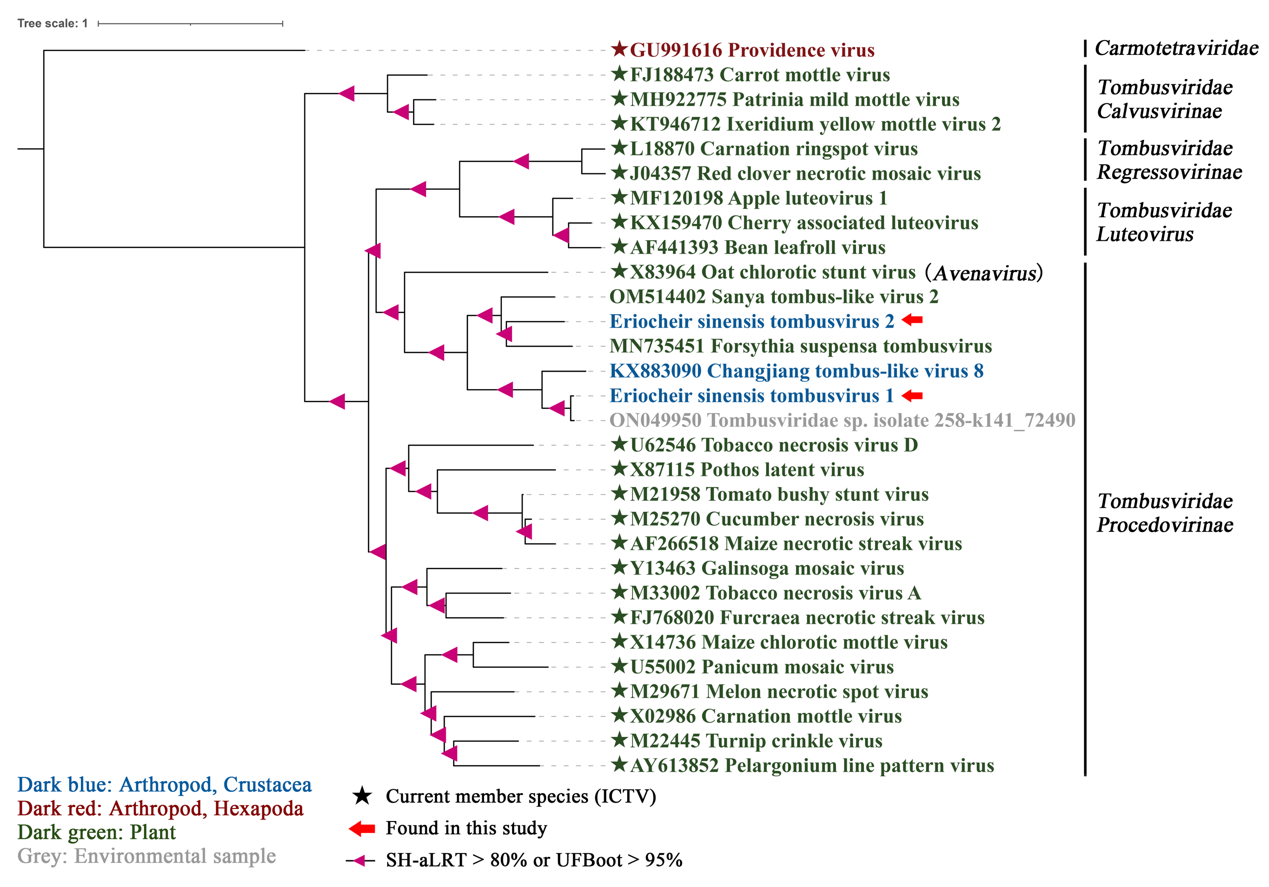


**Supplementary Figure 7. Phylogenetic tree of the order *Tolivirales*.** A mid-point rooted maximum likelihood phylogenetic tree was constructed using IQ-TREE2 with amino acid sequences encoding RdRp. Branch supports were determined using the Shimodaira–Hasegawa approximate likelihood ratio test (SH-aLRT) and ultrafast bootstrap (UFBoot) approach with 1,000 replicates. Branches with high bootstrap supports are indicated by pink triangles (SH-aLRT > 80% or UFBoot > 95%). The hosts of viruses are denoted by different colors of virus names, and the ICTV-accepted members of families or genera are denoted by star symbols beside virus names. Viruses identified in this study are denoted by red arrows beside virus names.


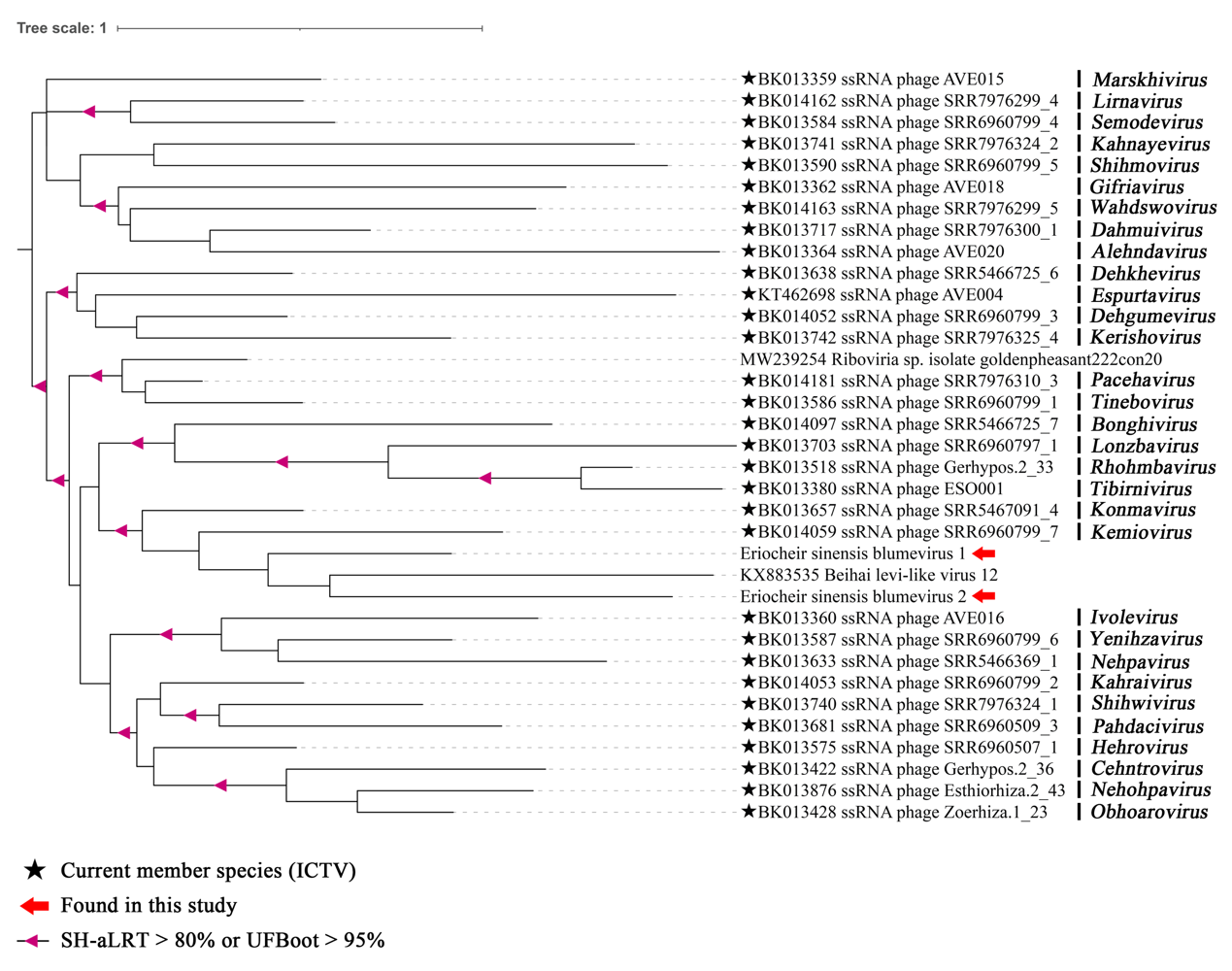


**Supplementary Figure 8. Phylogenetic tree of the order *Timlovirales*.** A mid-point rooted maximum likelihood phylogenetic tree was constructed using IQ-TREE2 with amino acid sequences encoding RdRp. Branch supports were determined using the Shimodaira–Hasegawa approximate likelihood ratio test (SH-aLRT) and ultrafast bootstrap (UFBoot) approach with 1,000 replicates. Branches with high bootstrap supports are indicated by pink triangles (SH-aLRT > 80% or UFBoot > 95%). The ICTV-accepted members of families or genera are denoted by star symbols beside virus names. Viruses identified in this study are denoted by red arrows beside virus names.


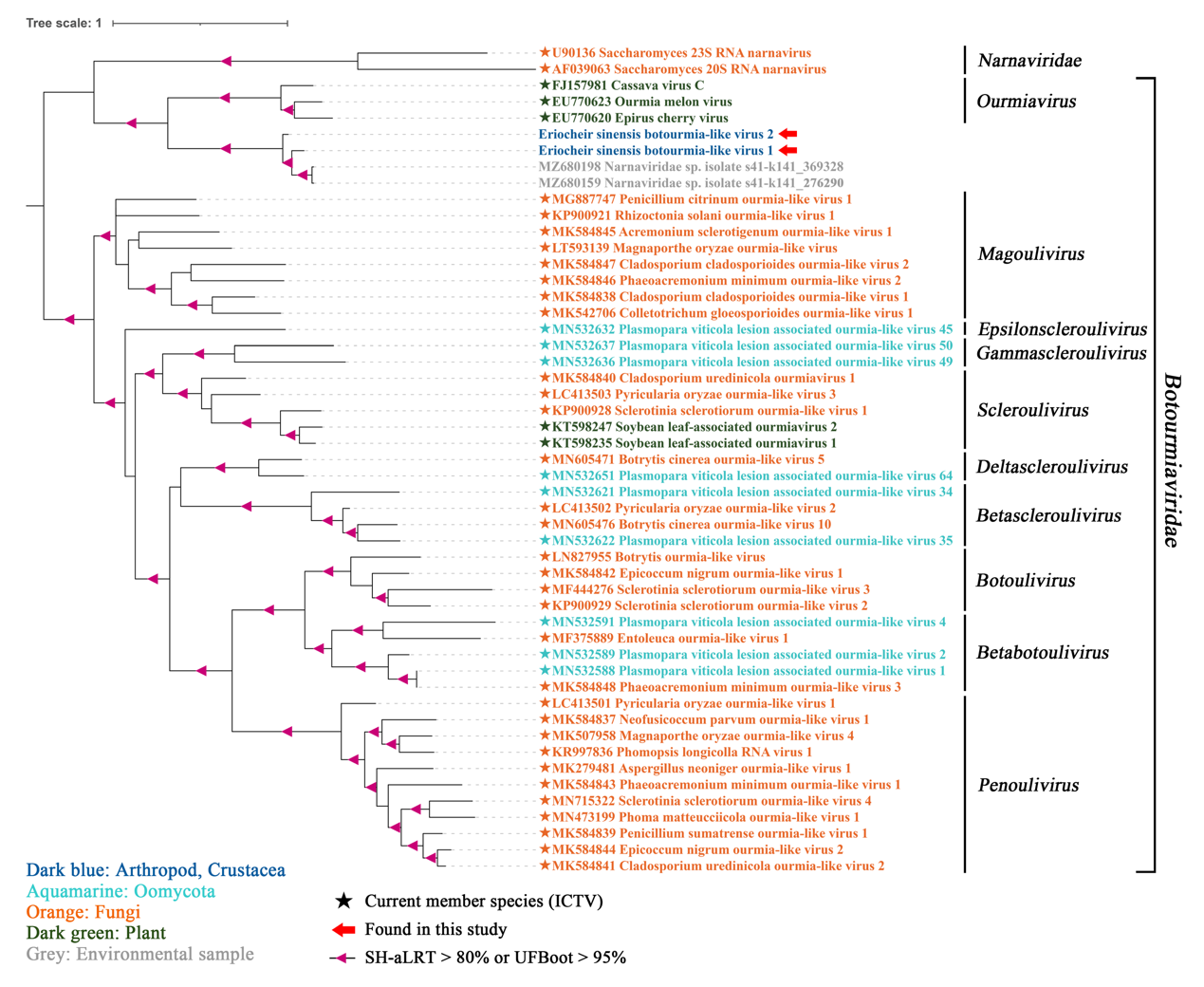


**Supplementary Figure 9. Phylogenetic tree of the order *Ourlivirales*.** A mid-point rooted maximum likelihood phylogenetic tree was constructed using IQ-TREE2 with amino acid sequences encoding RdRp. Branch supports were determined using the Shimodaira–Hasegawa approximate likelihood ratio test (SH-aLRT) and ultrafast bootstrap (UFBoot) approach with 1,000 replicates. Branches with high bootstrap supports are indicated by pink triangles (SH-aLRT > 80% or UFBoot > 95%). The hosts of viruses are denoted by different colors of virus names, and the ICTV-accepted members of families or genera are denoted by star symbols beside virus names. Viruses identified in this study are denoted by red arrows beside virus names.


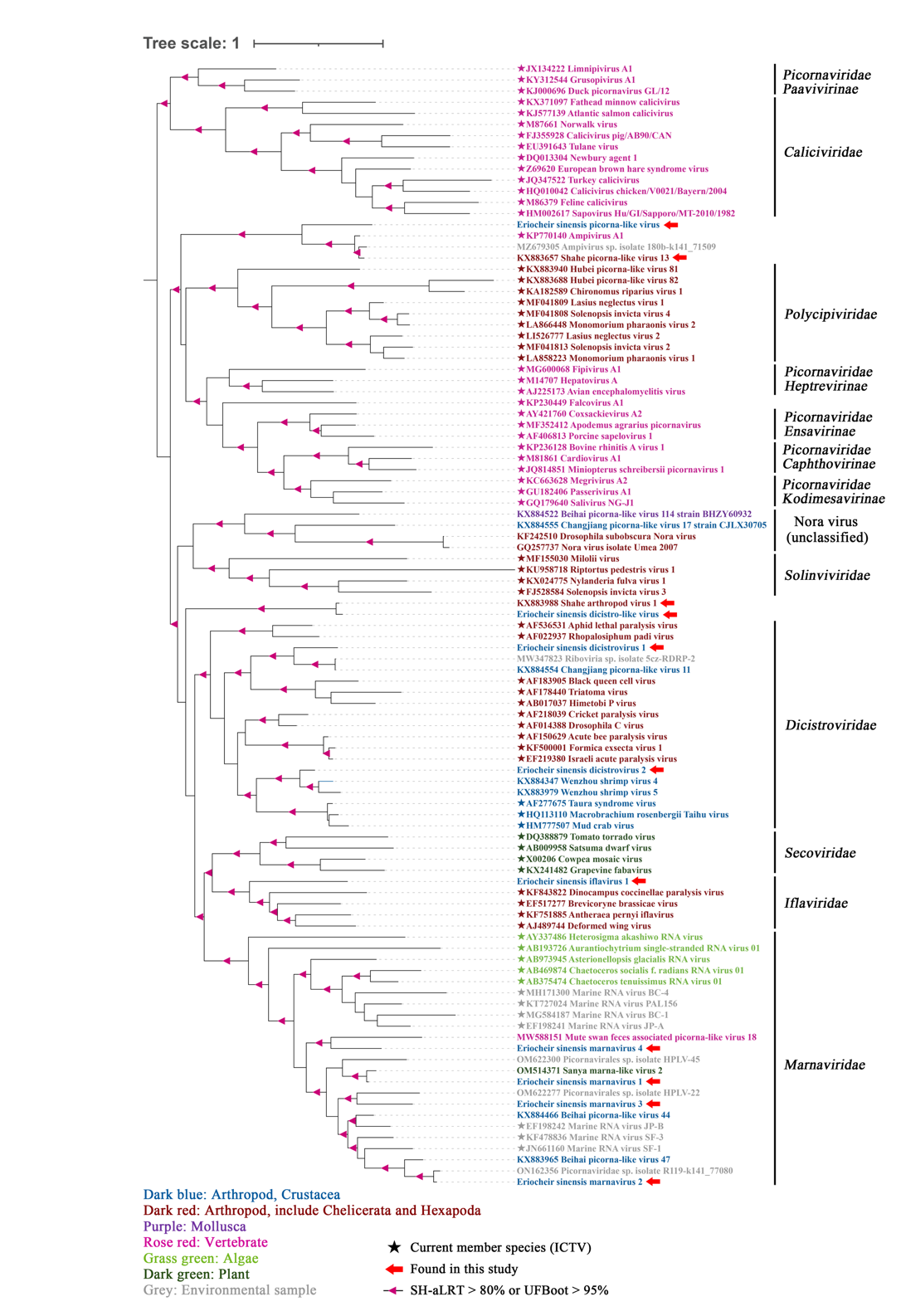


**Supplementary Figure 10. Phylogenetic tree of the order *Picornavirales*.** A mid-point rooted maximum likelihood phylogenetic tree was constructed using IQ-TREE2 with amino acid sequences encoding RdRp. Branch supports were determined using the Shimodaira–Hasegawa approximate likelihood ratio test (SH-aLRT) and ultrafast bootstrap (UFBoot) approach with 1,000 replicates. Branches with high bootstrap supports are indicated by pink triangles (SH-aLRT > 80% or UFBoot > 95%). The hosts of viruses are denoted by different colors of virus names, and the ICTV-accepted members of families or genera are denoted by star symbols beside virus names. Viruses identified in this study are denoted by red arrows beside virus names.


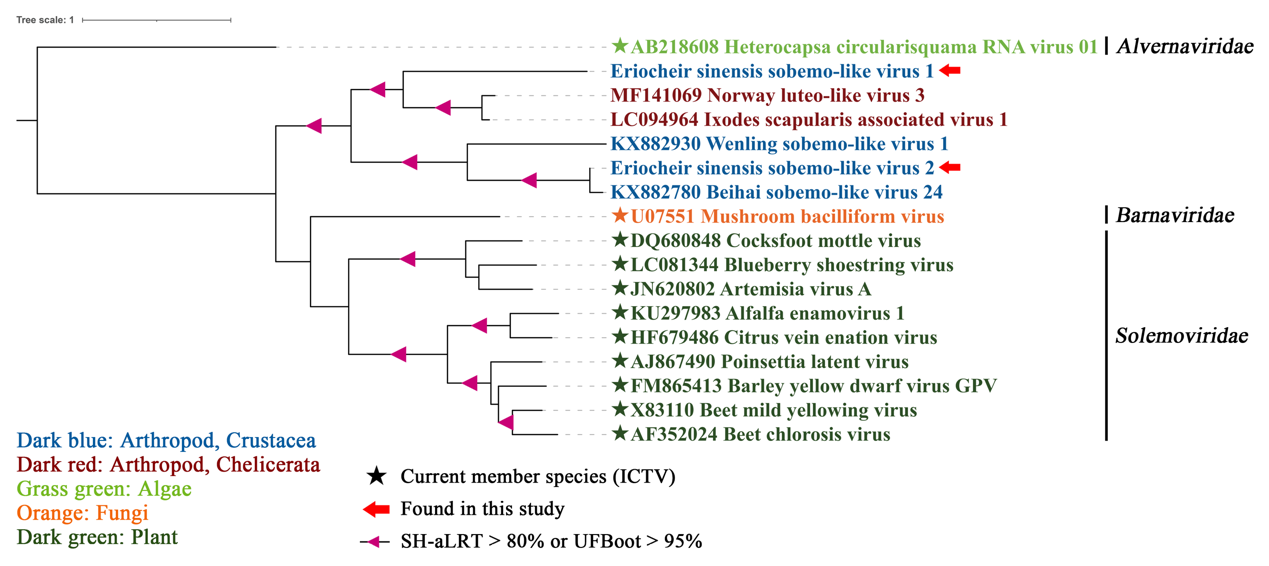


**Supplementary Figure 11. Phylogenetic tree of the order *Sobelivirales*.** A mid-point rooted maximum likelihood phylogenetic tree was constructed using IQ-TREE2 with amino acid sequences encoding RdRp. Branch supports were determined using the Shimodaira–Hasegawa approximate likelihood ratio test (SH-aLRT) and ultrafast bootstrap (UFBoot) approach with 1,000 replicates. Branches with high bootstrap supports are indicated by pink triangles (SH-aLRT > 80% or UFBoot > 95%). The hosts of viruses are denoted by different colors of virus names, and the ICTV-accepted members of families or genera are denoted by star symbols beside virus names. Viruses identified in this study are denoted by red arrows beside virus names.
